# The Vagus Nerve–Macrophage Connection: Tuning the Rheostat of Regenerative Medicine

**DOI:** 10.3390/ijms27198496

**Published:** 2026-09-23

**Authors:** Alexandre Henrique Caetano de Parma, João Protásio Netto, André Kruel, Luyddy Pires, Tomás Mosaner, Daniel Jorge, Stephany Huber, José Fábio Lana

**Affiliations:** 1 Department of Orthopedics, Brazilian Institute of Regenerative Medicine (BIRM), Indaiatuba 13334-170, SP, Brazil; kruel.andre@gmail.com (A.K.); luyddypires@gmail.com (L.P.); tomas@altodaspalmeiras.com.br (T.M.); danfjorge@gmail.com (D.J.); ioc.orthoregen@gmail.com (S.H.); josefabiolana@gmail.com (J.F.L.); 2 Regenerative Medicine, Orthoregen International Course, Indaiatuba 13334-170, SP, Brazil; 3 Department of Orthopedics and Traumatology, Federal University of Tocantins (UFT), Palmas 77001-090, TO, Brazil

**Keywords:** α7 nicotinic acetylcholine receptor, cholinergic anti-inflammatory pathway, macrophage polarization, NLRP3 inflammasome, matrix metalloproteinases, mesenchymal stromal cells

## Abstract

Chronic musculoskeletal disorders, including osteoarthritis, rheumatoid arthritis, and tendinopathies, converge on persistent synovial inflammation, matrix catabolism, and impaired repair. This review synthesizes molecular evidence that the cholinergic anti-inflammatory pathway (CAP), the efferent vagal arm of the inflammatory reflex, behaves as a rheostat of the musculoskeletal microenvironment. Activation of the α7 nicotinic acetylcholine receptor (α7nAChR) on macrophages, chondrocytes, osteoblasts, and synoviocytes suppresses nuclear factor-κB (NF-κB) and NLRP3 inflammasome signaling, downregulates MMP-1, MMP-3, and MMP-13, and reduces osteoblast RANKL production; effects on OPG and on the RANKL/OPG ratio under vagal stimulation remain uncharacterized, while favoring a pro-resolution M2-like macrophage program; context-dependent pro-inflammatory responses have also been reported. Bidirectional CAP and mesenchymal stromal cell (MSC) crosstalk indicates that MSCs both sense cholinergic cues and require an intact vagal arc for full immunomodulation. Along the gut–vagus–joint axis, dysbiosis and endotoxin translocation attenuate vagal signaling and amplify systemic inflammatory tone. Because orthobiologics such as platelet-rich plasma, bone marrow aspirate concentrate, and MSCs act within this milieu, cholinergic tone appears associated with the terrain that conditions their efficacy. Heart rate variability stratification and vagal neuromodulation may represent testable adjuncts, although prospective validation in orthobiologic populations remains absent. Preclinical and early clinical evidence is critically appraised throughout.

## 1. Introduction

Chronic musculoskeletal conditions, encompassing osteoarthritis (OA), rheumatoid arthritis (RA), tendinopathies, and chronic non-specific musculoskeletal pain, represent a leading cause of disability worldwide and impose a substantial and growing burden on individuals, healthcare systems, and economies [1,2]. Despite their clinical heterogeneity, these conditions share a convergent pathobiological substrate: persistent low-grade inflammation, progressive extracellular matrix (ECM) degradation, impaired mesenchymal stromal cell (MSC) function, and a dysregulated tissue repair response that collectively undermine the efficacy of both conventional and regenerative therapeutic strategies [3,4]. At the molecular level, this substrate is increasingly resolved into a limited set of recurring nodes, among them nuclear factor-κB (NF-κB) and NLRP3 inflammasome signaling, the transcriptional induction of matrix metalloproteinases (MMPs), and the receptor activator of NF-κB ligand to osteoprotegerin (RANKL/OPG) ratio that governs osteoclastogenesis. The present review examines how a single, anatomically defined neuroimmune circuit converges on precisely these nodes and thereby sets the molecular tone of the musculoskeletal microenvironment.

The inflammatory processes central to musculoskeletal pathology are not autonomous. They are subject to continuous bidirectional regulation by the autonomic nervous system, and in particular by the parasympathetic efferent arm represented by the vagus nerve. The Cholinergic Anti-Inflammatory Pathway (CAP), first characterized by Tracey and colleagues in the early 2000s, operates through efferent vagal fibers that reach the coeliac-mesenteric plexus and, from there, engage the splenic nerve. The final limb of this circuit is not a direct cholinergic vagal synapse on the splenic macrophage. The splenic nerve is predominantly catecholaminergic, and noradrenaline released from its terminals acts on β2-adrenergic receptors expressed by a specialized population of choline acetyltransferase (ChAT)-positive T lymphocytes resident in the spleen; it is the acetylcholine released by these lymphocytes that engages α7 nicotinic acetylcholine receptors (α7nAChR) on adjacent macrophages, suppressing the release of tumor necrosis factor-α (TNF-α), interleukin-1β (IL-1β), and high-mobility group box protein 1 (HMGB1) [5,6,7]. The precise neuroanatomy of this relay, in particular the extent of direct vagal input to the splenic nerve, remains under active investigation. This neuroimmune circuit functions as a physiological rheostat, continuously calibrating the magnitude of the inflammatory response in proportion to the intensity and resolution of the injurious stimulus.

In the context of chronic musculoskeletal disease, this regulatory capacity is frequently compromised. Attenuated vagal tone, evidenced by reduced heart rate variability (HRV) and diminished CAP efficiency, is consistently reported across RA and OA, and it correlates with elevated systemic inflammatory markers and worsened clinical outcomes [8,9,10]. The resulting sympathetic dominance sustains macrophage M1 polarization, perpetuates ECM catabolism through upregulated MMPs, and progressively degrades the tissue microenvironment required for effective endogenous repair and orthobiologic engraftment [11,12]. The loss of cholinergic restraint is therefore not a peripheral epiphenomenon of chronic disease but a candidate upstream contributor to the catabolic molecular program that defines it.

An additional dimension of complexity has emerged with the recognition of the gut–vagus–joint axis, a functionally integrated circuit in which intestinal dysbiosis attenuates vagal afferent signaling, elevates circulating lipopolysaccharide (LPS), and amplifies systemic inflammatory tone, thereby indirectly accelerating musculoskeletal deterioration [13,14]. Causal dependence of this circuit on vagal integrity has been demonstrated experimentally, in that microbiota-induced behavioural and inflammatory phenotypes were abolished by vagotomy, establishing the vagus nerve as an obligatory relay rather than a passive correlate of gut-derived signalling [15]. This systemic perspective reframes musculoskeletal disease not as a localized organ pathology but as one manifestation of a broader neuroimmune and metabolic dysregulation amenable to upstream, axis-level therapeutic intervention. It also predicts that interventions directed exclusively at the joint compartment will address only the distal end of a longer regulatory chain.

Against this background, a translational frontier is emerging in which vagal neuromodulation, delivered through bioelectronic stimulation, pharmacological α7nAChR agonism, microbiome-directed strategies, or the strategic coupling of orthobiologic therapies with CAP activation, may help restore the immunological conditions required for effective musculoskeletal regeneration [16,17,18]. This review synthesizes current mechanistic and translational evidence on the role of the vagus nerve and the CAP in governing the musculoskeletal microenvironment, with particular attention to macrophage M1/M2 reprogramming, ECM homeostasis, MSC biology, and the molecular consequences of restoring cholinergic tone. The literature is organized so that receptor-level evidence, tissue-level evidence, and systemic autonomic evidence are examined in sequence, allowing the strength of each layer to be assessed independently. The review closes by delimiting what the current evidence supports, what it does not yet support, and which experiments would resolve the difference. The multilevel architecture of this pathway, from its brainstem efferent origin to its convergent molecular effects on macrophage phenotype, extracellular matrix turnover, the mesenchymal stromal cell niche and the available therapeutic entry points, is summarized in Figure 1, in which panels A to C present the neural axis, macrophage reprogramming and matrix preservation, and panels D to F present the regenerative axis, the gut-to-joint axis and the therapeutic modalities.

### Literature Search Strategy

The evidence synthesized here was identified through iterative searches of PubMed/MEDLINE, Scopus, and Web of Science, with the final search conducted in 2026, complemented by backward and forward citation tracking of retrieved articles and by hand-searching the reference lists of previous reviews on neuroimmune regulation of inflammation. The search window ran from 2001, the year of the earliest primary report retained in this review, to July 2026, when the final search was executed, with preferential inclusion of work published in the last decade; earlier reports were retained when they constituted the primary description of a mechanism still in use. Of the 123 references cited, 95 (77%) were published in 2016 or later, and the median year of publication is 2021. Search terms were organized into six concept blocks: (i) pathway, comprising “cholinergic anti-inflammatory pathway”, “inflammatory reflex”, “vagus nerve stimulation”, and “vagal tone”; (ii) receptor, comprising “α7 nicotinic acetylcholine receptor”, “α7nAChR”, and “CHRNA7”; (iii) musculoskeletal disease, comprising “osteoarthritis”, “rheumatoid arthritis”, “tendinopathy”, “synovitis”, “cartilage”, and “subchondral bone”; (iv) molecular effectors, comprising “macrophage polarization”, “NF-κB”, “NLRP3”, “matrix metalloproteinase”, “RANKL”, “osteoprotegerin”, and “ADAMTS”; (v) regenerative agents, comprising “mesenchymal stromal cell”, “platelet-rich plasma”, “bone marrow aspirate concentrate”, and “orthobiologic”; and (vi) systemic modifiers, comprising “heart rate variability”, “gut microbiota”, “dysbiosis”, and “lipopolysaccharide”. Within each block, terms were combined with OR, using controlled vocabulary where the database offered it and free-text equivalents otherwise; blocks were combined with AND, pairing a pathway or receptor block with one of the remaining blocks in each iteration rather than executing a single exhaustive string. The verbatim strings were not archived at the time of searching, so the strategy is reported here at the level of the concept blocks. Peer-reviewed original research, systematic reviews, and meta-analyses in English were eligible when they addressed molecular or translational aspects of cholinergic immunomodulation in musculoskeletal tissue, or in systems whose mechanisms are directly transferable to it; conference abstracts, non-peer-reviewed preprints, and records without accessible full text were not used. Because several statements in this review report the absence of evidence, the criterion governing transfer from non-musculoskeletal systems is stated here: a mechanism was treated as transferable only when the receptor or signaling node in question had been demonstrated in a musculoskeletal cell type or tissue in at least one independent report, so that the transferred evidence bore on the regulation of an already-established target rather than on the existence of the target itself. Statements of the form ‘no study identified in this review’ should be read as referring to this search window and to the terms listed above, and not as a claim of exhaustive coverage. This is a narrative review: no protocol was registered, no PRISMA workflow or formal risk-of-bias appraisal was applied, and article selection reflected the authors’ assessment of mechanistic relevance, so a degree of selection bias cannot be excluded.

Because this review draws on evidence of unequal strength, and because the distance between a molecular observation and a clinical inference is where a framework of this kind is most easily overread, we classify the supporting evidence into four tiers and use them consistently in the sections that follow. Tier I is evidence obtained directly in musculoskeletal cells, tissue, or patients. Tier II is evidence obtained in other biological systems where the receptor or signaling node has independently been shown to operate in musculoskeletal tissue, so that the transferred finding bears on the regulation of an established target. Tier III is associative human evidence, principally the correlations between autonomic indices and circulating inflammatory markers. Tier IV is hypothesis-generating extrapolation to orthobiologic outcomes, for which no direct evidence exists. Table 1 assigns each principal claim of the review to one of these tiers. The central proposition of the review, that cholinergic tone conditions the biological terrain in which orthobiologics act, is Tier IV, and no argument developed here upgrades it.

## 2. The Macrophage as a Central Effector: M1/M2 Reprogramming by the Cholinergic Anti-Inflammatory Pathway

### 2.1. The M1/M2 Framework in Musculoskeletal Inflammation

In the pathophysiology of chronic musculoskeletal disease, macrophage polarization has emerged as one of the most mechanistically tractable therapeutic targets. Synovial macrophages are abundant resident immune cells that oscillate between a pro-inflammatory M1 phenotype, characterized by secretion of TNF-α, IL-1β, IL-6, and a broad repertoire of MMPs and ADAMTS proteases, and an anti-inflammatory, reparative M2 phenotype, which produces IL-10, transforming growth factor-β (TGF-β), and mediators that support cartilage protection and tissue repair [12,19,20,21,22]. In osteoarthritis, M1 macrophage dominance correlates with disease severity, synovitis grade, and cartilage degradation scores, with M1-derived R-spondin-2 identified as a direct driver of chondrocyte apoptosis and bone erosion [23,24,25,26]. These observations position the macrophage as the principal cellular relay through which upstream neural signals could be translated into structural outcomes at the joint.

**Figure 1:**
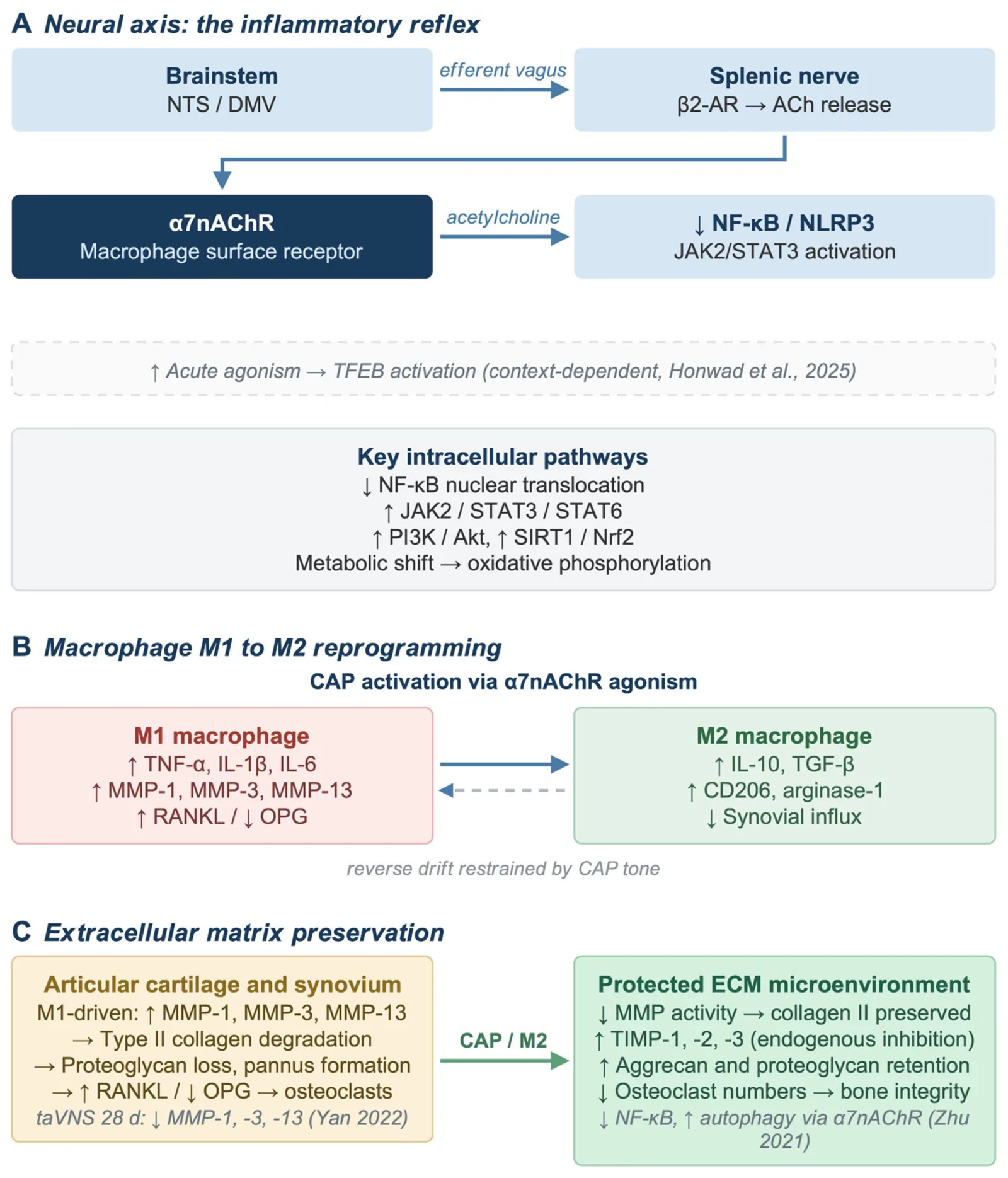

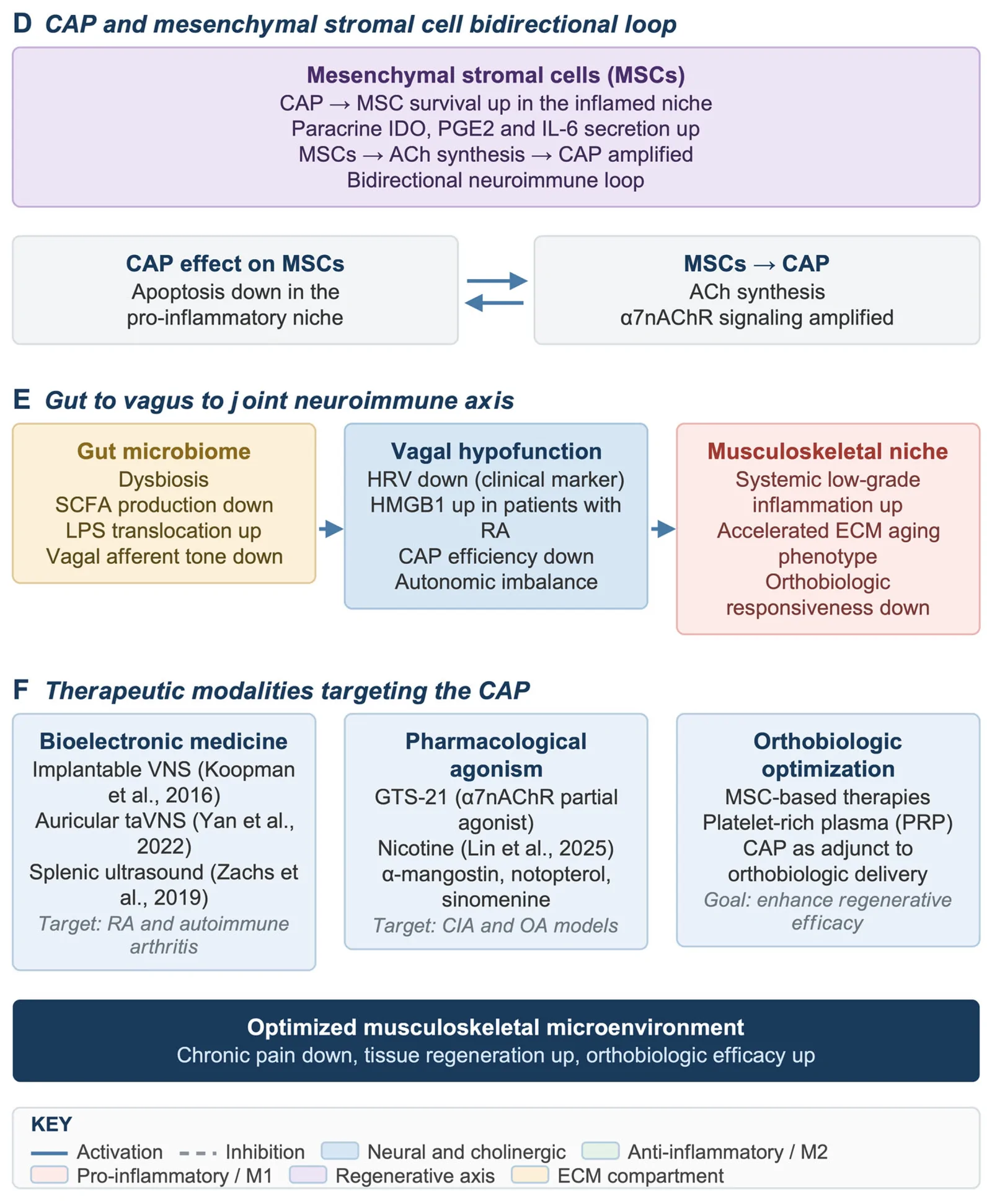
Molecular architecture of the cholinergic anti-inflammatory pathway (CAP) as a rheostat of the musculoskeletal microenvironment: neural axis, macrophage reprogramming and matrix preservation. (**A**) Efferent vagal output from the brainstem reaches the splenic nerve, where beta2-adrenergic signaling is coupled to acetylcholine availability; alpha7nAChR engagement on macrophages is associated with JAK2/STAT3 activation and with reduced NF-kappaB and NLRP3 activity, while the dashed inset records the opposing observation that acute agonism may induce TFEB-dependent responses in a context-dependent manner [27]. (**B**) M1 to M2 reprogramming, with the intracellular mediators represented in the panel listed in the adjacent box; the dashed return arc denotes reverse drift restrained by cholinergic tone rather than a demonstrated rate. (**C**) Cholinergic protection of the extracellular matrix (ECM), from the M1-driven catabolic state to a protected compartment with preserved collagen II, increased tissue inhibitors of metalloproteinases and reduced osteoclastogenesis [23]. Solid connectors denote activation and dashed connectors inhibition; colours follow the key at the foot of the second page of this figure. The SIRT1 and Nrf2 nodes, the oxidative phosphorylation shift, indoleamine 2,3-dioxygenase and short-chain fatty acid production orient the reader to mechanisms reported in the cited literature but not developed in this review. All relationships were derived by the authors exclusively from the cited primary literature; the diagram was drawn as a vector schematic, with computational assistance used for layout and typesetting. (**D**) Bidirectional loop between the CAP and mesenchymal stromal cells (MSCs), in which cholinergic tone is associated with reduced MSC apoptosis in a pro-inflammatory niche while MSC-derived acetylcholine may in turn amplify alpha7nAChR signaling. (**E**) The gut, vagus and joint axis, linking dysbiosis to reduced short-chain fatty acid production, increased lipopolysaccharide translocation and attenuated vagal afferent tone, and from there to a musculoskeletal niche with low-grade systemic inflammation and an accelerated matrix ageing phenotype. (**F**) Therapeutic modalities that engage the pathway, grouped as bioelectronic, pharmacological and orthobiologic, converging on an optimized microenvironment. Inclusion in panel (**F**) denotes that a modality engages the pathway and not that it is an established clinical strategy; nicotine and the selective agonists appear as mechanistic probes, for the reasons given in Section 3.2. The key at the foot applies to all panels of Figure 1.

A terminological note applies to the whole of what follows. We retain M1 and M2 as heuristic shorthand for the extremes of a functional continuum, and where the distinction matters, we prefer pro-inflammatory or catabolic and pro-resolution or reparative to describe the states themselves, for the reasons developed in Section 2.4. The balance between these states is not static; it represents a dynamic equilibrium continuously modulated by cytokine gradients, pattern recognition receptor engagement, and, critically, autonomic neuroimmune signals. The CAP constitutes the most physiologically relevant of these autonomic inputs. Its disruption, through vagotomy, autonomic neuropathy, or chronic sympathetic dominance, predictably shifts the macrophage equilibrium toward M1 polarization and perpetuates a cytokine environment hostile to tissue repair [8,9,10]. The molecular corollary is that cholinergic input acts less as an on and off switch than as a modifier of the transcriptional thresholds at which macrophages commit to an inflammatory program.

### 2.2. α7nAChR-Mediated M2 Polarization: Mechanistic Evidence

The molecular basis for CAP-mediated macrophage reprogramming centers on α7nAChR signaling. A mechanistically rigorous study using both THP-1 cells and primary human monocyte-derived macrophages demonstrated that α7nAChR activation with the selective agonist PNU-282987 significantly enhanced M2 markers, including CD206 and IL-10, whereas α7nAChR deficiency reduced M2 proportions at inflammatory sites in vivo, which is consistent with a direct, cell-autonomous M2-polarizing role for this receptor [28]. Complementary evidence from gut macrophage models showed that α7nAChR agonism increases STAT6 activation and M2 surface markers while suppressing M1 cytokines in RAW264.7 macrophages, with effects abrogated by selective antagonism [29]. In the central nervous system, microglial conversion from M1 to M2 following α7nAChR activation occurs through JAK2/STAT3 and PI3K/Akt pathways, indicating that the polarizing mechanism is conserved across tissue macrophage populations [30].

An important caveat to the uniform M2-polarizing narrative has been raised. Acute α7nAChR activation in murine macrophages was shown to induce TFEB-mediated lysosomal biogenesis and a transient pro-inflammatory response under specific stimulation conditions, indicating that the directionality of the cholinergic polarization signal is context-dependent [27]. This directionality may be modulated by the baseline activation state of the macrophage, by agonist concentration, and by receptor desensitization kinetics, three variables that differ substantially between in vitro protocols and therapeutic exposure in vivo. This finding underscores the need for precise dosimetry in therapeutic protocols targeting the CAP, and it argues against treating α7nAChR agonism as a uniformly anti-inflammatory manoeuvre.

### 2.3. Evidence in Arthritis and Joint-Specific Macrophage Models

In the context of inflammatory arthritis, CAP activation by multiple pharmacological approaches has consistently reduced synovial macrophage M1 activity and improved disease outcomes. α-Mangostin, a natural α7nAChR agonist and CAP activator, attenuated adjuvant-induced arthritis in rats through the CAP and SIRT1 pathway in macrophages, reducing M1 markers and synovial inflammation [31,32]. Notopterol, a phytochemical with α7nAChR agonist properties, attenuated synovitis via α7nAChR-dependent metabolic reprogramming of macrophage polarization, reducing NF-κB activation and M1 cytokine secretion [33]. GTS-21, a selective α7nAChR partial agonist, attenuated collagen-induced arthritis by inhibiting peripheral monocyte trafficking into the synovium, effectively reducing the cellular substrate for M1 polarization at the disease site [34].

Bioelectronic approaches have yielded converging evidence. Neurostimulation of the CAP via vagal nerve stimulation ameliorated disease in the rat collagen-induced arthritis model through macrophage-mediated suppression of TNF-α and downstream inflammatory cascades [35]. Non-invasive ultrasound stimulation of the spleen in inflammatory arthritis produced comparable anti-arthritic effects by activating the splenic neural arc of the CAP [36]. Pharmacological stimulation of nicotinic acetylcholine receptors attenuated collagen-induced arthritis (CIA) in mice, and α7nAChR-deficient animals developed more severe disease, which supports the interpretation that endogenous cholinergic tone constitutes a physiological brake on articular inflammation [37,38].

The CAP also modulates the cellular composition of the synovial inflammatory infiltrate beyond macrophages. In CIA models, CAP activation through Chinese herbal formulas modulated dendritic cell differentiation and reduced adaptive immune activation [39]. In RA patients, attenuated CAP activity, measured by serum HMGB1 as a surrogate, correlated with disease activity indices, providing clinical evidence that the pathway is biologically active and clinically relevant in this population [8]. The cholinergic anti-inflammatory pathway has been reviewed as a broadly relevant therapeutic target across autoimmune rheumatic diseases, with α7nAChR expression documented in synovial tissue, peripheral immune cells, and circulating monocytes of RA patients [40,41,42,43].

### 2.4. The M1/M2 Paradigm: Heuristic Value and Biological Complexity

The M1/M2 binary framework, as employed throughout this review, functions as a conceptually tractable scaffold for understanding macrophage immunobiology but does not accurately represent the full complexity of macrophage activation states in vivo. Single-cell transcriptomic analyses and tissue-specific phenotyping have consistently revealed that synovial macrophages in OA and RA occupy a continuous spectrum of activation states, with cells co-expressing markers historically attributed to distinct M1 or M2 poles and transitioning fluidly between functional identities in response to local cytokine gradients, metabolic inputs, and mechanical cues [12,19,20,25]. The archetypical M1 phenotype (LPS and IFN-γ polarized) and M2 phenotype (IL-4 and IL-13 polarized) derived from in vitro culture conditions represent the extremes of this spectrum rather than its operative reality in diseased joints. Within this framework, the therapeutic goal of CAP activation is better conceptualized not as a binary phenotypic conversion from M1 to M2 but as a re-calibration of macrophage functional output along the inflammatory and reparative continuum, incrementally reducing pro-inflammatory cytokine secretion and MMP expression while enhancing pro-resolution mediator production and phagocytic clearance capacity.

This distinction has practical implications for therapeutic design. A transient initial inflammatory macrophage response is required for normal tissue repair initiation, including the platelet-derived growth factor (PDGF) and vascular endothelial growth factor (VEGF) release that facilitates angiogenesis and progenitor cell recruitment [24,44]. The objective of CAP-based co-therapy is therefore not to suppress this physiologically necessary phase but to prevent its pathological amplification and persistence, which amounts to a re-tuning rather than an abolition of macrophage inflammatory competence. Framed in these terms, the relevant therapeutic question is one of timing and amplitude rather than of simple suppression.

## 3. The Extracellular Matrix as a Target: Cholinergic Protection Against Proteolytic Catabolism

### 3.1. ECM Catabolism in Musculoskeletal Disease

The extracellular matrix of articular cartilage, synovial tissue, bone, tendon, and skeletal muscle constitutes not merely a structural scaffold but a dynamic regulatory substrate that stores growth factors, transmits mechanical signals, and governs cell fate decisions. Its progressive degradation by matrix metalloproteinases (MMPs) and by members of the a disintegrin and metalloproteinase with thrombospondin motifs (ADAMTS) family is among the most consequential pathological events in inflammatory and degenerative musculoskeletal disease [45]. In arthritis, MMP-1, MMP-3, MMP-9, MMP-13, ADAMTS-4, and ADAMTS-5 mediate the cleavage of type II collagen and aggrecan, the two principal load-bearing components of hyaline cartilage, generating a self-perpetuating cycle of structural deterioration and inflammatory amplification [45,46]. Mature articular cartilage is avascular, aneural, and populated by a sparse, largely post-mitotic chondrocyte population, so the anabolic capacity available to rebuild a cleaved collagen network is severely constrained and even a modest but sustained proteolytic excess accumulates as effectively irreversible structural loss [24].

Synovial fibroblast-like synoviocytes (FLS) represent a critical cellular driver of this catabolic cascade. In RA, FLS adopt an invasive, hyperproliferative phenotype driven by cytokine stimulation, predominantly IL-1β and TNF-α, that upregulates MMP secretion, promotes synovial pannus formation, and invades and destroys periarticular cartilage and bone [47]. Chondrocytes participate autonomously in the same process, since on exposure to IL-1β they suppress the anabolic program sustaining type II collagen and aggrecan synthesis while inducing the enzymes that degrade both, so the cell responsible for matrix maintenance becomes an agent of its destruction [23,48]. The modulation of these two populations by the cholinergic system has attracted mechanistic interest, and it appears to operate at the level of transcriptional control rather than of individual catalytic domains.

### 3.2. α7nAChR Activation Suppresses MMP Expression and Cartilage Degradation

Direct evidence for a chondroprotective role of α7nAChR signaling has been established in multiple experimental models. In a rat OA model, the selective α7nAChR agonist PNU-282987 significantly attenuated cartilage degeneration, reducing MMP-1 and MMP-13 expression in chondrocytes while enhancing autophagy-mediated cellular protection, with structural preservation confirmed histologically [23]. Nicotine, acting via α7nAChR in chondrocytes, attenuated IL-1β-induced upregulation of HMGB1, TLR4, and NF-κB/NLRP3 signaling, a pathway driving both cytokine amplification and MMP transcription, which offers a mechanistic account of the anti-catabolic effect [48]. Broader α7nAChR activation in OA chondrocytes inhibits NF-κB/NLRP3 inflammasome activation and enhances autophagy, mitigating OA progression in animal models [23].

Four caveats apply to the compounds discussed above, and grouping them as CAP-targeting interventions should not obscure the distances between them. Receptor selectivity differs by orders of magnitude: PNU-282987 and GTS-21 were developed as selective α7 ligands and function principally as mechanistic probes, whereas nicotine is a non-selective nicotinic agonist acting at α4β2 and other heteromeric subtypes in addition to α7, so an effect observed with nicotine cannot be attributed to α7 engagement without pharmacological dissection. The α7 receptor desensitizes rapidly and with agonist-dependent kinetics, which means that sustained exposure does not translate into sustained signaling and that dosing schedules, not merely doses, determine the biological result; partial agonists such as GTS-21 and positive allosteric modulators were developed partly in response to this problem. Systemic tolerability separates the compounds further: nicotine carries cardiovascular, addictive, and pro-neoplastic liabilities that make it untenable as a chronic therapeutic strategy in this population, and it should be read here strictly as an experimental tool that helped establish receptor involvement in chondrocytes. Finally, the phytochemical agonists discussed above act at multiple targets beyond α7nAChR, so their anti-catabolic effects in joint tissue cannot be ascribed to the CAP on the basis of the available data. Of the pharmacological approaches surveyed, none has advanced to a musculoskeletal clinical trial, and the realistic near-term candidates for translational evaluation are the bioelectronic modalities rather than any of these compounds.

In synovial FLS, the α7nAChR is constitutively expressed and was among the first demonstrations of a functional cholinergic receptor on non-neuronal joint cells [47]. Subsequent work in FLS indicated that cholinergic signaling reduces NF-κB-dependent cytokine transcription and MMP secretion, functionally opposing the pannus-forming, matrix-destructive phenotype that characterizes active RA [49]. In the subchondral bone compartment, murine osteoblasts also express α7nAChR, and receptor activation dampens IL-1β-induced IL-6, MMP-3, and RANKL production [46,50]. The result is a reduction in osteoclast activation signals and in subchondral remodeling, a critical contributor to joint structural failure in OA [50].

The cholinergic system in the joint, which includes not only neuronal innervation but also non-neuronal acetylcholine synthesis by synoviocytes, chondrocytes, and immune cells, has been characterized as a locally active anti-inflammatory and anti-catabolic network [46]. Activation of this pathway in preclinical OA models appears to attenuate cartilage degradation and pain behavior in association with reduced MMP-9 and cytokine levels, supporting the plausibility of joint-level CAP modulation as a structure-modifying strategy [46]. Transcutaneous auricular vagus nerve stimulation (taVNS) in RA rat models reduced both bone and cartilage destruction scores, indicating that systemic vagal activation translates into local ECM protection [51]. Sinomenine, which increases α7nAChR expression and inhibits NF-κB in FLS and adjuvant-induced arthritis, further suggests that pharmacological upregulation of receptor expression, rather than transient activation alone, may be a viable long-term approach to ECM preservation [49].

### 3.3. Transcriptional Control of Matrix-Degrading Enzymes: NF-κB and AP-1 as Convergent Nodes

The breadth of the cholinergic effect on matrix turnover is most coherently explained at the level of transcription. IL-1β and TNF-α activate the IκB kinase complex, which licenses proteasomal degradation of IκB-α and releases NF-κB dimers for nuclear translocation, while the ERK, JNK, and p38 mitogen-activated protein kinase cascades stabilize the Fos and Jun components of activator protein 1 (AP-1) [52]. The promoter and enhancer regions of the inducible MMP genes, including MMP-1, MMP-3, MMP-9, and MMP-13, contain functional κB and AP-1 response elements [53], so the two families act combinatorially rather than redundantly and maximal induction requires concurrent occupancy. This architecture accounts for a recurring observation in the cholinergic literature, namely that interventions reducing NF-κB activation in chondrocytes, synoviocytes, or macrophages produce coordinated rather than enzyme-selective reductions in proteolytic output [23,48,49]. Three pharmacological lines converge on this node: nicotine suppressed the HMGB1/TLR4/NF-κB module in chondrocytes and, with it, the downstream catabolic program [48]; sinomenine increased α7nAChR expression while inhibiting NF-κB in FLS and in adjuvant-induced arthritis [49]; and notopterol reduced NF-κB activation and M1 cytokine secretion through α7nAChR-dependent metabolic reprogramming of macrophage polarization [33]. Read together, these findings suggest that α7nAChR signaling acts as a transcriptional brake upstream of the proteolytic network rather than as an inhibitor of any individual enzyme, which may account for the breadth of structural protection reported across preclinical models [23,46,51]. The distinction matters practically, since broad-spectrum catalytic MMP inhibition has proven difficult in musculoskeletal disease whereas upstream transcriptional restraint spares the constitutive component of matrix turnover [45]. Whether α7nAChR activation also constrains the AP-1 arm, for instance through JAK2/STAT3-dependent interference with JNK signaling of the kind described in microglia [30], has not been examined in joint tissue.

### 3.4. Tissue Inhibitors of Metalloproteinases and the Stoichiometry of Matrix Protection

Net matrix turnover is determined not by protease abundance alone but by the molar relationship between active enzymes and their endogenous inhibitors, the tissue inhibitors of metalloproteinases (TIMP-1 to TIMP-4). TIMPs occlude the catalytic zinc site of their target enzymes through their N-terminal domain, forming essentially irreversible one-to-one complexes, and TIMP-3 is distinctive in associating with the matrix itself and in inhibiting the aggrecanases ADAMTS-4 and ADAMTS-5 as well as MMPs [54]. Degenerative and inflammatory joint disease is consequently better characterized as a disturbance of the MMP/TIMP ratio than as a simple increase in proteolysis, since cytokine-driven MMP induction typically outpaces any compensatory inhibitor response and the resulting imbalance permits sustained cleavage even at modest enzyme concentrations [11,45]. This framing bears on how cholinergic interventions should be read: the reductions in MMP-1, MMP-3, MMP-9, and MMP-13 reported after α7nAChR activation or vagal stimulation [23,48,51] indicate movement toward matrix preservation, but the available musculoskeletal studies report protease expression almost uniformly without measuring TIMPs in parallel. Whether cholinergic protection operates by suppressing protease transcription alone or additionally by relieving cytokine-mediated inhibitor suppression therefore cannot be resolved, and the two mechanisms carry different implications for dosing and durability. Reporting MMP/TIMP ratios with activity-based assays would strengthen the mechanistic case at low cost within existing preclinical designs.

### 3.5. Aggrecanases ADAMTS-4 and ADAMTS-5 and the Early Loss of Aggrecan

Aggrecan depletion precedes overt collagen network failure in the natural history of cartilage degeneration [55], and this temporal sequence assigns particular pathogenic weight to the aggrecanases ADAMTS-4 and ADAMTS-5 [45,46]. Both enzymes cleave the aggrecan core protein within the interglobular domain, releasing glycosaminoglycan-bearing fragments into synovial fluid [56] and stripping the tissue of the fixed negative charge that generates its osmotic swelling pressure and compressive stiffness. Because the resulting loss of hydration alters the strain distribution borne by the residual collagen network, aggrecan cleavage is mechanically as well as biochemically permissive of subsequent collagenolysis, placing the aggrecanases upstream of the MMP-13-driven events that dominate later disease. ADAMTS-5 is the dominant aggrecanase in murine models [57,58] whereas the relative contribution of the two enzymes in human disease remains contested [56], and both are induced by IL-1β and TNF-α through NF-κB and AP-1 dependent transcription. That shared dependency predicts that the cholinergic brake described in Section 3.3 should also constrain aggrecanase expression, yet no study identified in this review measured ADAMTS-4 or ADAMTS-5 expression, aggrecan neoepitope generation, or synovial fluid aggrecan fragment concentrations after α7nAChR agonism or vagal stimulation in a musculoskeletal model. Because aggrecanase-mediated proteoglycan loss is an early and initially reversible event, this is among the most clearly defined gaps in the CAP literature on joint tissue, and one closable with models already in routine use.

### 3.6. The RANKL/OPG Axis and Subchondral Bone Remodeling

Structural failure of the joint is not confined to cartilage. Subchondral remodeling, osteophyte formation, and periarticular erosion are executed by osteoclasts whose differentiation and survival depend on receptor activator of nuclear factor-κB ligand (RANKL), presented by osteoblasts, osteocytes, synovial fibroblasts, and activated T cells, and antagonized by the soluble decoy receptor osteoprotegerin (OPG). Engagement of RANK recruits TRAF6 and activates NF-κB together with NFATc1, the master regulator of osteoclast differentiation [59], so the RANKL/OPG ratio rather than either molecule alone governs net resorption. Inflammatory cytokines shift this ratio unfavorably, with IL-1β, TNF-α, and IL-6 increasing RANKL availability while suppressing OPG. The same NF-κB activation that drives MMP transcription therefore also drives osteoclastogenesis, making NF-κB the shared node coupling cartilage catabolism to bone resorption during inflammatory bone loss and impaired repair [44,60].

The cholinergic evidence at this node is direct but narrow. Murine osteoblasts express α7nAChR, and receptor activation dampened IL-1β-induced IL-6, MMP-3, and RANKL production, reducing the osteoclast-activating signal available to the subchondral compartment and attenuating age-related osteoarthritic change [50]. At the tissue level, taVNS reduced bone and cartilage destruction scores in rats with collagen-induced arthritis [51], and α7nAChR subunit knockout animals developed more severe arthritis than wild-type controls, indicating that endogenous cholinergic tone constrains structural damage as well as synovial inflammation [38]. These observations appear consistent with a model in which cholinergic tone lowers RANKL availability and restrains osteoclastogenesis at the osteochondral junction, complementing the chondrocyte-level effects described above and offering a unified account of why CAP activation may protect both compartments. The OPG arm has received almost no attention: whether α7nAChR activation raises OPG expression, and whether the circulating or synovial fluid RANKL/OPG ratio responds to vagal stimulation in patients, remains unreported and would be straightforward to assess within neuromodulation trials already under way in inflammatory joint disease [61,62].

## 4. The Regenerative Axis: Mesenchymal Stromal Cells, the Cholinergic System, and the Osteoimmune Niche

### 4.1. The Immunological Dependence of Regenerative Outcomes

Effective musculoskeletal regeneration requires more than the delivery of competent progenitor cells or bioactive growth factors; it demands a local immune environment that is permissive for cell engraftment, angiogenesis, matrix anabolism, and differentiated function. Experimental models of bone and cartilage repair have consistently demonstrated that an early, transient inflammatory phase, mediated by M1 macrophages releasing PDGF and VEGF, is required for normal healing initiation [24,44]. Persistence of M1 dominance beyond the first 72 h, however, actively impairs bone formation and generates fibrocartilage rather than hyaline repair tissue [60,63]. The transition from an M1 to an M2 macrophage-dominated environment is therefore not merely permissive but mechanistically required for the establishment of a pro-regenerative osteoimmune niche, in which MSCs receive the molecular signals, predominantly IL-10, TGF-β, and IL-4, necessary for osteogenic and chondrogenic differentiation [64,65,66,67].

Sustained M1 activity reverses this process. Chronic inflammation alters MSC differentiation, driving fibrocartilage formation over hyaline repair, impairing osteogenesis, and reducing MSC viability through caspase-mediated apoptosis in high-TNF-α environments [64,68]. Orthobiologic therapies, including MSCs, PRP, BMAc, exosomes, and biomaterial scaffolds, operate within this inflammatory milieu and are directly modified by it [63,64]. Inappropriate timing or chronicity of the host inflammatory response can therefore convert a regenerative intervention into a neutral or harmful one [69].

### 4.2. MSC-CAP Bidirectional Crosstalk

An underappreciated dimension of MSC biology is the bidirectional relationship between MSCs and the CAP. On one axis, MSCs are recipients of cholinergic signals: they express a functional cholinergic system including choline acetyltransferase (ChAT), vesicular acetylcholine transporter (VAChT), and multiple nAChR and mAChR subunits including α7 and M2 muscarinic receptors [70,71]. In human MSCs, α7-containing nAChR mediate nicotine-induced Ca2+ influx; physiological agonist concentrations modulate migration without impairing viability, while high doses induce apoptosis, indicating a dose-dependent sensitivity that is directly relevant to therapeutic design [72]. Selective M2 muscarinic activation inhibits MSC proliferation and migration in adipose-derived MSCs, suggesting that the full cholinergic receptor repertoire shapes MSC behavior in a receptor subtype-specific manner [73].

On the reciprocal axis, MSCs are potent activators of the CAP, although the evidence for this direction of the interaction derives entirely from non-musculoskeletal disease models and is classified as Tier II throughout this review. In acute respiratory distress syndrome models, infused MSCs upregulated lung ChAT and VAChT expression, increased local acetylcholine concentrations, and required an intact vagus nerve and functional α7nAChR for their full anti-inflammatory therapeutic effect, demonstrating that MSC immunomodulation is mechanistically coupled to CAP engagement rather than being fully autonomous [74]. MSC-derived prostaglandin E2 (PGE2) promotes acetylcholine synthesis and release as a downstream mechanism, providing a molecular bridge between MSC paracrine signaling and neuroimmune regulation [74]. In sepsis models, Wharton’s jelly-derived MSCs reduced leukocyte infiltration, cytokines, and organ apoptosis through α7nAChR-dependent mechanisms, with effects abolished by selective antagonism [75]. Both observations were made in acute systemic inflammation rather than in joint tissue, and no equivalent experiment has been reported in an intra-articular or periarticular setting, so the requirement for an intact vagal arc should be read as a property demonstrated in those models and proposed, not shown, for musculoskeletal applications of MSCs.

This bidirectional relationship has substantial implications. The inflammatory milieu that impairs MSC function is precisely the milieu that attenuates CAP activity, so restoring CAP function may improve the conditions under which MSC therapies operate. The CAP suppresses the TNF-α, IL-1β, and IL-6 that impair MSC osteogenesis, and MSCs delivered into a CAP-active environment may in turn amplify local cholinergic tone [70,74,76]. Should both arms operate concurrently in vivo, the result would be a self-reinforcing regenerative circuit rather than two independent anti-inflammatory effects.

### 4.3. Orthobiologics and the Cholinergic Microenvironment

The clinical translation of the CAP-MSC interaction to orthobiologic practice involves several converging lines of evidence. PRP, through its growth factor payload (PDGF, TGF-β, VEGF, IGF-1), exerts direct effects on macrophage polarization and tissue repair, but its efficacy appears to be conditioned by the host inflammatory state [77]. The macrophage polarization state of the recipient tissue therefore emerges as a modifiable variable rather than a fixed patient characteristic, raising the possibility that the immunological context of orthobiologic delivery may itself be conditioned by adjunct interventions [64,69]. MSC-mediated immune modulation in the bone marrow and systemic circulation has been shown to suppress cytokine storms and regulate T-cell and NK-cell activity through paracrine mechanisms that include CAP activation [69,76].

MSCs have also been proposed as a therapeutic tool for neuroinflammation-related pathology, with their capacity to modulate cholinergic signaling implicated as part of the reparative mechanism [78]. This conceptual bridge, from peripheral musculoskeletal regeneration to central neuroimmune regulation, positions MSCs and the CAP as elements of a single therapeutic framework rather than independent modalities. The corresponding research priority is to design orthobiologic delivery protocols that account for this neuroimmune context rather than treating it as background noise. Doing so requires specifying the molecular content of that context, which the following subsections address.

### 4.4. IL-10 and TGF-β Signaling in Osteogenic and Chondrogenic Commitment

The assertion that an M2-polarized environment is permissive for MSC differentiation acquires mechanistic content only when the mediators and their intracellular routes are specified. IL-10 signals through the IL-10 receptor complex and the JAK1/STAT3 axis to induce SOCS3, which restrains MyD88 and NF-κB-dependent transcription of TNF-α, IL-1β, and IL-6 [79]. The consequence for MSCs is largely indirect but functionally decisive, because this withdraws the cytokine input that suppresses osteogenic transcriptional programs and drives caspase-mediated apoptosis in progenitor populations [64,68]. Experiments using macrophage-conditioned medium support this interpretation, in that the osteogenic capacity of human MSCs varies with the activation state of the macrophages that conditioned the medium [68]. MSC fate is therefore instructed by the surrounding immune phenotype rather than merely tolerated by it, and the reciprocal MSC-immune interaction in bone injury has been described as a determinant of repair quality rather than an incidental feature of the healing field [64].

TGF-β occupies a more ambivalent position in this circuit. Signaling through TGF-β receptor II and the Smad2/3 axis, it is required for chondrogenic commitment and for induction of SOX9, the transcription factor governing COL2A1 and ACAN expression [80]. It also contributes to the immunosuppressive repertoire through which MSCs restrain effector T cell activity and promote regulatory T cell induction [69]. Sustained or excessive TGF-β activity, however, is associated with fibrotic matrix deposition [81] and with the type I collagen rich fibrocartilage that characterizes failed cartilage repair, which is precisely the histological outcome reported when M1 dominance persists in experimental repair models [24,44,64]. The therapeutic implication is that the objective of CAP-based conditioning is not maximal anti-inflammatory cytokine exposure but temporal control: an IL-10 and TGF-β enriched window of sufficient amplitude and duration to permit engraftment and lineage commitment, followed by resolution rather than by chronic profibrotic signaling. Strategies that deliberately shape the early immunoregulatory environment, including engineered extracellular vesicles and inflammatory priming of MSCs before delivery, indicate that this window is experimentally tractable [66,67]. Its kinetics under cholinergic modulation remain undefined, since intra-articular IL-10 and TGF-β concentrations have not been measured during vagal stimulation in a musculoskeletal setting.

### 4.5. The Cholinergic Receptor Repertoire of Mesenchymal Stromal Cells

The capacity of MSCs to participate in cholinergic signaling rests on an expression profile considerably broader than the α7 subunit alone. MSCs derived from bone marrow and from adipose tissue express both nicotinic and muscarinic acetylcholine receptors, and receptor engagement elicits functional responses rather than merely detectable transcripts [70,71]. They also carry the machinery required for autocrine and paracrine acetylcholine handling, including ChAT and VAChT, which places MSCs among the non-neuronal populations capable of synthesizing and releasing the ligand to which they themselves respond [70,71,74]. This dual competence, as both source and target of acetylcholine, is what makes the bidirectional crosstalk described in Section 4.2 mechanistically coherent rather than merely correlative.

Functionally, the receptor subtypes are not interchangeable, and this heterogeneity has been underestimated in translational discussions of cholinergic modulation. α7-containing nicotinic receptors mediate nicotine-induced calcium influx and modulate MSC migration, with agonist concentrations in the physiological range altering migratory behavior without compromising viability while higher concentrations induce apoptosis, a dose dependence with direct implications for protocol design [72]. Selective M2 muscarinic activation, by contrast, inhibits proliferation and migration in adipose-derived MSCs [73]. The aggregate cholinergic effect on a delivered MSC population will therefore depend on which subtypes predominate and on the local acetylcholine concentration achieved, neither of which is characterized for the preparations used in clinical practice. Two consequences follow: the cholinergic phenotype of an MSC-containing product is in principle a characterizable release attribute, reportable alongside the surface marker and viability metrics that currently define product identity, although no standardized assay exists; and because bone marrow aspirate concentrate and adipose-derived preparations differ in tissue origin and cellular composition, their receptor profiles should not be assumed equivalent [82,83,84].

### 4.6. MSC Senescence and the Senescence-Associated Secretory Phenotype

The regenerative competence of the MSC pool is not a fixed property, and its decline provides a further point of contact between the CAP and orthobiologic outcomes. Replicative and stress-induced senescence in MSCs is accompanied by proliferative arrest [85], reduced osteogenic and chondrogenic differentiation capacity, and acquisition of a senescence-associated secretory phenotype (SASP) [86] comprising pro-inflammatory cytokines such as IL-6 and IL-8, chemokines including monocyte chemoattractant protein 1, and matrix-degrading enzymes including MMP-3. The SASP is transcriptionally driven in substantial part by NF-κB, which establishes a self-amplifying circuit in which inflammatory signaling promotes senescence, senescent cells secrete further inflammatory and catabolic mediators, and neighboring cells are recruited into the same phenotype through paracrine spread [87]. In the aging and chronically inflamed skeleton this circuit is associated with impaired bone healing, sustained M1 macrophage activation, increased osteoclast activity, and suppressed osteoblast formation and MSC osteogenic potential [88]. The catabolic output of the SASP therefore overlaps with the proteolytic program of Section 3, suggesting that senescence and matrix degradation are two expressions of one inflammatory transcriptional state.

The relevance for regenerative practice is uncomfortable but direct. Bone marrow aspirate concentrate and adipose-derived preparations are harvested from precisely the patients in whom this circuit is most active, namely older individuals with long-standing degenerative joint disease, so the delivered cell population may itself contain a partially senescent, SASP-competent fraction whose secretory output opposes the intended therapeutic effect [63,77,88]. A mechanistic connection to the CAP is plausible but unproven: if NF-κB is the shared transcriptional node driving both the SASP and the catabolic program of Section 3, then α7nAChR-mediated NF-κB suppression [23,48] might be expected to attenuate SASP output, and priming strategies that modify MSC immunomodulatory potency before delivery provide a practical experimental route to test this [67]. No study identified in this review examined whether α7nAChR agonism or vagal stimulation alters senescence markers or SASP secretion in MSCs. Resolving this would determine whether CAP modulation acts only on the host niche or also on the therapeutic cell product itself.

## 5. Vagal Tone as a Biological Rheostat for Orthobiologic Efficacy: A Temporal Framework

### 5.1. The Regenerative Microenvironment as a Prerequisite, Not a Bystander

The clinical success of orthobiologic therapies, including PRP, BMAc, and MSCs, has historically been evaluated as a function of product composition, that is, platelet concentration, cell viability, and growth factor content. Emerging evidence reframes this paradigm, in that biological host factors and the local inflammatory state of recipient tissue are increasingly recognized as primary determinants of response variability [77,89]. In a mechanistically significant study, Zahir et al. demonstrated that the in vitro anti-inflammatory capacity of patient-derived PRP in a macrophage and fibroblast coculture model correlated with patient-reported clinical outcomes in knee osteoarthritis, which suggests that it is not merely what is injected, but the biological context into which it is delivered, that governs efficacy [90]. This observation shifts attention from product standardization toward host characterization.

This principle reorients the research question. If the local and systemic inflammatory milieu conditions orthobiologic success, then any intervention capable of modulating that milieu becomes, by definition, a candidate therapeutic co-strategy. The CAP, tonically regulated by vagal efferent activity, fulfills precisely this role. Meta-analytic and prospective data consistently demonstrate that higher HRV, indexing stronger vagal activity, is inversely associated with circulating C-reactive protein (CRP), IL-6, and fibrinogen across multiple populations [7,91,92,93,94,95,96]. Longitudinal analyses further indicate that lower vagally mediated HRV predicts higher CRP concentrations years later, suggesting that baseline vagal tone is associated with, and may help regulate, long-term inflammatory trajectories [96]. These associations extend beyond cardiovascular populations: in patients with COVID-19, lower vagal indices correlated with higher IL-6 and CRP, greater oxygen requirements, and longer hospitalization [97]; in bladder cancer, sympathovagal imbalance was linked to elevated systemic inflammation and impaired adaptive immunity [98]; and in neovascular macular degeneration, measurable deficits in vagal tone were associated with failed resolution of systemic inflammatory activation [99].

In the musculoskeletal context specifically, vagotomy in a rat model of knee OA accelerated pain-related gait alterations and worsened cartilage thinning and subchondral bone remodeling, providing direct preclinical evidence that low vagal tone is not merely permissive of joint pathology but may be actively contributory [100]. The logical corollary is clinically substantial, in that patients presenting for orthobiologic therapy with low vagal tone may be delivering their biological product into a systemically hostile terrain, one characterized by persistent macrophage M1 polarization, NF-κB-driven matrix catabolism, and impaired MSC osteogenic differentiation [77,88]. This inference is mechanistic rather than empirical, because no study has yet measured vagal indices in a cohort receiving orthobiologic treatment. The distinction between plausibility and demonstration is maintained throughout the remainder of this section.

### 5.2. Before the Injection: Conditioning the Biological Terrain

The concept of biological terrain conditioning, that is, preparing the recipient tissue and systemic environment prior to orthobiologic delivery, currently has no established protocol in regenerative medicine practice. The mechanistic rationale, however, is coherent. Chronic low-grade systemic inflammation, or inflammaging, disrupts bone healing by sustaining macrophages in a pro-inflammatory M1 phenotype, increasing osteoclast activity, and suppressing osteoblast formation and MSC osteogenic potential [88]. Senescent MSCs within this milieu compound the problem, secreting pro-inflammatory and anti-regenerative factors that sustain a cycle of regenerative failure [77,88], a dynamic equally relevant to BMAc-based therapies, whose heterogeneous outcomes in clinical series may partly reflect this immunological substrate [82,83,101,102].

Pre-operative vagal tone has already demonstrated predictive value for post-surgical inflammatory trajectories in orthopedic settings. Gitler et al. showed that lower pre-operative HRV was associated with higher postoperative CRP, slower inflammatory resolution, and longer hospital stay following total knee replacement [103]. Liu et al. reported that pre-operative HRV predicted postoperative blood inflammatory biomarker levels in surgical patients [104]. Hildenborg et al. found that distinct pre-operative HRV patterns were associated with divergent neuroimmune inflammatory trajectories following abdominal surgery [105]. Extrapolating to the orthobiologic context, a patient with autonomic imbalance may arrive at the procedure with a systemically primed inflammatory state that is mechanistically antagonistic to the reparative goals of PRP or BMAc.

Transcutaneous vagus nerve stimulation (tVNS) offers a plausible, non-invasive pre-conditioning strategy. In a randomized controlled trial, Elsehrawy et al. reported that tVNS significantly reduced pain, improved functional scores, and attenuated pro-inflammatory cytokines in patients with knee OA, with effects sustained beyond the treatment period [61]. Cruz et al. proposed neuromodulation as a potentially disease-modifying strategy in OA, arguing that autonomic dysfunction contributes mechanistically to disease progression and that bioelectronic approaches could function as adjuncts to conventional joint-focused therapies [106]. If a structured pre-procedural course of tVNS were capable of shifting the macrophage phenotype toward M2, reducing synovial NF-κB activity, and attenuating subchondral bone inflammatory remodeling, as the mechanistic evidence from α7nAChR studies would predict [23,48,50], then the injected orthobiologic would encounter a more receptive biological environment. This conditional formulation is deliberate, because the intermediate molecular endpoints have not been measured in tVNS-treated human joints.

### 5.3. During and After Delivery: Sustaining the Regenerative Window

The biological activity of orthobiologics unfolds over days to weeks and is sensitive to the cytokine balance within the target tissue. PRP initiates a cascade of growth factor signaling mediated by PDGF, TGF-β, VEGF, and IGF-1, whereas BMAc delivers a heterogeneous mixture of MSCs, hematopoietic progenitors, and bioactive mediators whose differentiation and immunomodulatory potential depend on the local microenvironment [77,82,83,84,107,108]. The OA joint expresses a complete non-neuronal cholinergic system, and α7nAChR activation in chondrocytes and osteoblasts directly suppresses IL-1β-induced IL-6, MMP-3, RANKL, and NF-κB and NLRP3 inflammasome activity [23,46,48,50]. Cholinergic signaling via α7nAChR also regulates the migration of monocyte-derived macrophages during acute inflammation, providing an additional mechanism by which vagal activity shapes the cellular composition of the post-procedural joint microenvironment [109].

A mechanistically underappreciated dimension concerns the interaction between the CAP and platelet biology. Bravo-Iniguez et al. demonstrated that cervical vagus nerve stimulation (VNS) primed platelets for enhanced α-granule release via a circuit requiring splenic T cells and platelet α7nAChR, increasing cytosolic calcium influx without inducing resting platelet activation [110]. Platelets themselves express functional α7nAChRs and store endogenous acetylcholine, forming an autocrine cholinergic amplification loop, and α7nAChR stimulation potentiates fibrinogen receptor activation in response to classical platelet agonists, while α7 antagonism abolishes this effect [111]. Megakaryocytes also express nicotinic acetylcholine receptor subunits including α7, suggesting that the cholinergic influence on platelet biology extends to the thrombopoietic stage [112]. Reviews further emphasize that α7 stimulation can modulate pro-inflammatory platelet reactivity within the broader CAP framework, although the specific effect on individual growth factor release profiles within PRP, namely PDGF, TGF-β, and VEGF, remains unquantified [113,114]. Two cautions follow, and we state them rather than leave them implicit. First, enhanced α-granule release or greater platelet responsiveness cannot be equated with improved regenerative efficacy: the same primed state could plausibly shift inflammatory, thrombotic, or reparative signaling in different directions depending on tissue context and timing of delivery. Second, the studies establishing cholinergic control of platelet function were not designed around orthobiologic preparations. We therefore present this as an experimentally testable mechanistic question, namely whether vagal conditioning measurably alters the growth factor content or release kinetics of an autologous platelet concentrate, and not as evidence that the CAP optimizes PRP.

In the post-procedural phase, the persistence of low vagal tone would be expected to re-establish the inflammatory conditions present at baseline, potentially eroding therapeutic gains achieved by orthobiologic delivery. The largely equivalent outcomes between PRP and BMAc at two years reported by Anz et al. [115], and the heterogeneous results observed across orthobiologic modalities in systematic evidence [116,117,118,119], may in part reflect the return of systemic inflammatory dysregulation in patients whose autonomic baseline was never addressed. This interpretation is one of several compatible with the data, and it competes with explanations based on product heterogeneity, injection technique, and patient phenotype at baseline. It is presented here because it is testable with existing, non-invasive instrumentation rather than because the current evidence favors it over the alternatives.

### 5.4. An Integrative Model: Vagal Tone as a Stratification and Co-Therapeutic Variable

Synthesizing the evidence across these temporal phases, a coherent integrative model can be articulated in which vagal tone, measurable non-invasively as HRV, may function simultaneously as a patient stratification variable and a modifiable co-therapeutic target in the context of orthobiologic therapy. In such a model, pre-procedural HRV assessment would be used to identify patients with cardiac autonomic imbalance and, alongside circulating inflammatory markers, to characterize baseline inflammatory burden. We use HRV here as an index of cardiac vagal modulation, not as a measurement of α7nAChR-dependent inflammatory reflex activity in the joint, and the constraints on that inference are set out in Section 6.4. Under this proposed stratification, which we advance as an experimental design rather than as a clinical pathway, patients with low HRV together with elevated CRP or IL-6 would constitute the group in which neuromodulatory pre-conditioning could be tested prior to orthobiologic delivery, aligned with the reported capacity of tVNS to reduce OA-related inflammation and pain within clinically relevant timeframes [61,120]. It should be emphasized that this stratification and co-therapeutic model remains to be prospectively validated in orthobiologic populations specifically: although its components are individually grounded in preclinical and cross-disease clinical evidence (Section 5.1, Section 5.2 and Section 5.3), the direct link between pre-procedural vagal tone and orthobiologic treatment outcomes has not yet been empirically tested and should be regarded as a hypothesis-generating framework rather than an established clinical pathway. The interventions that engage this pathway are compared in Table 2, which lists each modality with its primary mechanism, downstream effects and clinical application, while the evidence tier and the outstanding translational gap of each are given in Table 3.

Concurrent vagal modulation, sustained through tVNS or HRV-augmenting lifestyle interventions such as structured aerobic exercise, slow-paced breathing, or mindfulness-based stress reduction, would aim to maintain an M2-polarized macrophage microenvironment and α7nAChR-mediated matrix protection during the critical window of growth factor signaling and cellular engraftment that follows orthobiologic delivery. Post-procedural HRV monitoring would provide a dynamic, non-invasive window into the patient’s regenerative microenvironment, potentially enabling early detection of inflammatory rebound before clinical deterioration becomes manifest. This model does not require new molecules or invasive procedures; it requires a reconceptualization of orthobiologic therapy as a biological event embedded within an autonomic and neuroimmune context, one that may be actively shaped to maximize the therapeutic signal. The absence of prospective clinical data directly testing this framework defines the principal opportunity for the next generation of translational research in regenerative musculoskeletal medicine.

The components of the framework outlined in this review rest on separable bodies of evidence, and it is worth stating at the outset which of them are established and which are not. Bioelectronic access to the CAP arc has been demonstrated in human inflammatory disease; its application as a pre-procedural conditioning strategy for orthobiologic therapy has not. Bioelectronic activation of the CAP through implantable vagus nerve stimulation (iVNS) carries an established clinical track record in neurology and psychiatry, where it has been used as an approved therapy for drug-resistant epilepsy and for treatment-resistant depression, with tolerability characterized over extended follow-up in the psychiatric literature [121]. Regulatory approval dates and cumulative exposure figures were not verified against primary regulatory documentation for this review and are therefore not stated here. Its translation into inflammatory joint disease was directly demonstrated by Koopman et al. [62], who showed in a first-in-human trial that implantable VNS in patients with active rheumatoid arthritis significantly reduced disease activity scores and circulating TNF-α concentrations, confirming that the CAP arc is pharmacologically accessible through bioelectronic means in a human inflammatory joint disease. Vagal neuromodulation has since been evaluated in other inflammatory conditions, including a pilot study in Crohn’s disease that reported clinical and biological improvement [122]. Reports in post-operative inflammatory states and in COVID-19-associated cytokine dysregulation exist but were not systematically appraised here, and the strength of that evidence should not be inferred from the present summary. In the musculoskeletal context specifically, the randomized trial by Elsehrawy et al. in knee OA [61] and the preclinical evidence for tVNS in post-traumatic OA [120] indicate that non-invasive bioelectronic CAP modulation may produce clinically meaningful anti-inflammatory effects within the articular environment. What these data establish is that the CAP arc is pharmacologically and bioelectronically accessible in human inflammatory joint disease. They do not establish that engaging it before an orthobiologic procedure alters the outcome of that procedure, which is a distinct claim and remains untested. The framework advanced here is therefore an experimental hypothesis built on an accessible target, not a validated clinical strategy awaiting adoption.

## 6. Discussion

### 6.1. Mechanistic Convergence Across Levels of Analysis

The disorders grouped in this review differ enough that the conclusions should be read as stratified rather than uniform. The direct evidence for CAP engagement in joint tissue derives overwhelmingly from rheumatoid arthritis, where the inflammatory reflex was first shown to be pharmacologically accessible in humans, and from osteoarthritis, where receptor expression and anti-catabolic signaling have been demonstrated in chondrocytes and osteoblasts and where one randomized trial of non-invasive stimulation has been reported. For these two conditions the framework advanced here rests on a mechanistic base in the relevant tissue. Tendinopathy is different: the inflammatory contribution is contested, the tissue is comparatively hypocellular and poorly innervated, and we identified no study examining α7nAChR signaling in tenocytes, so any extension of the framework to tendon is extrapolation from other musculoskeletal tissues. Chronic non-specific musculoskeletal pain is different again, since the therapeutic target there is nociceptive and central rather than structural, and vagal neuromodulation has an analgesic rationale largely independent of the matrix-protective mechanisms reviewed here. Conflating an analgesic effect with a tissue-conditioning effect would be an error, and the predictions set out below concern the latter. Accordingly, the framework is best regarded as testable in inflammatory and degenerative joint disease, hypothesis-generating for tendon disorders, and mechanistically distinct from the analgesic applications in chronic non-specific pain.

The body of evidence synthesized in this review converges on a single, clinically consequential proposition: the vagus nerve and the CAP constitute a physiological axis whose tone appears to influence the permissiveness of the musculoskeletal microenvironment for repair. This proposition is not merely mechanistically plausible; it is supported by convergent lines of evidence spanning molecular receptor pharmacology, preclinical arthritis models, human autonomic physiology, and the emerging biology of orthobiologic interventions. What remains absent, and what this discussion identifies as the defining gap for translational research, is a prospective clinical trial that directly tests whether restoring cholinergic tone improves orthobiologic outcomes in patients with chronic musculoskeletal disease. Until such a trial is conducted, the framework should be read as an organizing hypothesis rather than as a clinical recommendation.

The mechanistic argument for CAP-mediated musculoskeletal regulation is comparatively robust by the standards of translational biology, in that it operates coherently across multiple levels of analysis. At the receptor level, α7nAChR activation on macrophages, chondrocytes, osteoblasts, and fibroblast-like synoviocytes suppresses overlapping catabolic and inflammatory signals, including NF-κB, NLRP3, MMP transcription, and RANKL, indicating that a single receptor class exerts broad structural protection across the principal cellular effectors of joint degradation [23,46,48,50]. At the tissue level, taVNS attenuates bone and cartilage destruction scores in CIA rat models [51], vagotomy accelerates OA pathogenesis [100], and pharmacological CAP activators including GTS-21, sinomenine, and α-mangostin reduce both synovitis and structural damage in experimental arthritis [31,32,34,49]. At the systemic level, HRV inversely predicts circulating inflammatory markers including CRP, IL-6, and fibrinogen across diverse populations [91,92,93,94,95,96], and lower pre-operative vagal tone predicts higher post-surgical inflammatory burden and delayed recovery in orthopedic settings [103,104,105].

The addition of the gut–vagus–joint axis introduces a further dimension of mechanistic coherence [13,14]. Intestinal dysbiosis does not merely alter local gut physiology; it attenuates vagal afferent signaling, elevates circulating LPS, and amplifies systemic inflammatory tone in a manner that may ultimately accelerate joint degradation. Specific taxonomic shifts have been described in inflammatory arthritis, with expansion of Prevotella copri correlating with new-onset untreated rheumatoid arthritis [123], although such associations identify candidate organisms rather than establishing that any single taxon drives the articular phenotype. This systemic reframing carries a clinically important implication, in that a patient presenting for intra-articular orthobiologic therapy may be carrying an upstream neuroimmune deficit that growth factor delivery alone would not correct. The strength of this argument nevertheless depends on evidence generated largely in rodent models of behaviour and systemic inflammation rather than in articular endpoints, and the inferential distance to the human joint should not be understated. The functional organization of this circuit, from intestinal barrier state through vagal afferent signaling to the joint compartment, together with the restorative interventions proposed to act upon it, is depicted in Figure 2, whose four panels present the intestinal microenvironment (Figure 2A), the neural axis (Figure 2B), the joint microenvironment (Figure 2C) and the proposed translational pipeline (Figure 2D).

### 6.2. The Biological Terrain Framework: Strengths and Limitations

The integrative model outlined in Section 5, in which vagal tone functions as a stratification variable and modifiable co-therapeutic target in the context of orthobiologic delivery, rests on mechanistic convergence rather than on direct empirical evidence. This distinction must be stated with precision. No published randomized controlled trial has prospectively assigned patients to a vagal pre-conditioning protocol prior to PRP, BMAc, or MSC injection and measured orthobiologic-specific outcomes. The critical inference, namely that the reported capacity of tVNS to reduce OA-related inflammation and pain [61,120] translates into enhanced orthobiologic efficacy, is mechanistically grounded but empirically unproven in this specific clinical context.

Similarly, while cholinergic signaling modulates α-granule release and potentiates platelet activation via α7nAChR [110,111], the downstream effect on the specific growth factor profile of PRP, including PDGF, TGF-β, VEGF, and IGF-1, has not been quantified. The possibility that CAP activation during PRP delivery could optimize growth factor output therefore represents a scientifically tractable question that the current literature does not resolve. A further unresolved variable is dosimetry, since the same receptor that mediates anti-inflammatory reprogramming can produce transient pro-inflammatory responses under specific stimulation conditions [27]. Any clinical protocol built on this framework would need to define stimulation parameters and agonist exposure with a precision that present data do not supply.

These limitations do not diminish the translational relevance of the framework; they define its experimental frontier. The heterogeneous outcomes reported across orthobiologic modalities in systematic evidence [116,117,118,119], and the broadly equivalent results between PRP and BMAc at two years [115], suggest that current protocols may be missing a variable of sufficient explanatory power to account for inter-patient variability. The biological terrain framework offers a candidate variable, namely vagal tone and its downstream inflammatory setpoint, that is measurable, potentially modifiable, and mechanistically connected to the biology of every orthobiologic currently in clinical use. Whether it accounts for a clinically meaningful fraction of that variability is an empirical question that remains open.

### 6.3. MSC-CAP Bidirectionality as a Therapeutic Opportunity

An underappreciated implication of the evidence reviewed in Section 4 concerns the bidirectional relationship between MSCs and the CAP. MSCs delivered into a CAP-active environment express functional α7nAChR and muscarinic receptors that modulate their migration, differentiation, and paracrine output [70,71,72,73]. Reciprocally, in models of acute respiratory distress syndrome and sepsis, infused MSCs upregulate local cholinergic markers including ChAT and VAChT, and their full anti-inflammatory effect was lost when the vagal arc was interrupted [74,75]. This dependence has therefore been demonstrated in systemic inflammatory models, not in bone, cartilage, tendon, or orthobiologic settings, and its transfer to the musculoskeletal compartment is an inference rather than an observation. This bidirectionality suggests that the CAP and MSC therapies are not merely compatible but mechanistically coupled, such that each may amplify the therapeutic potential of the other. Whether this coupling is exploitable in a clinical orthobiologic protocol, for example by combining tVNS with intra-articular MSC or BMAc injection, is a question that current evidence positions as tractable, and it is worth noting that the receptor-dependence data supporting it derive from pulmonary and septic rather than articular models.

### 6.4. Heart Rate Variability as a Clinical Tool: Promise and Current Boundaries

The proposal that pre-procedural HRV measurement should inform orthobiologic patient selection rests on a consistent body of autonomic physiology [91,92,96,103,104,105], but the distance between that literature and a usable clinical rule remains substantial. The supporting evidence is of three distinct kinds, and conflating them overstates the readiness of the measure. Meta-analytic and cohort work establishes an inverse association between vagally mediated HRV and circulating inflammatory markers across healthy and clinical populations [91,92], and longitudinal data indicate that this association is not merely contemporaneous, since lower HRV predicted higher C-reactive protein concentrations four years later in healthy non-smoking adults [96]. Perioperative studies add temporal ordering in a surgical setting, with pre-operative vagal activity associated with clinical outcomes after total knee replacement [103], pre-operative HRV predicting post-operative inflammatory biomarker levels [104], and distinct pre-operative HRV patterns accompanying divergent trauma-induced innate immune trajectories [105]. What none of these studies provide is an orthobiologic-specific outcome, so the inferential step from autonomic index to injectate response remains untested.

Four methodological constraints stand between this evidence and clinical use. First, HRV is not a single quantity but a family of time-domain, frequency-domain, and non-linear indices that carry different physiological meanings, and the literature summarized above does not converge on which index best tracks inflammatory burden in musculoskeletal disease. Second, recording conditions govern the numbers obtained, since posture, respiration, time of day, recording duration, and artifact-correction method all shift the values, and the absence of a common acquisition protocol across the cited studies limits cross-study comparability. Third, HRV is sensitive to variables that have nothing to do with the joint, including age, sex, physical fitness, body composition, sleep, medication with autonomic activity, and acute psychological state, so any threshold intended for orthopedic use would have to be adjusted for these covariates rather than applied as a raw cut-off. Fourth, the existing cut-offs derive predominantly from cardiovascular, oncological, and critical care populations [104,105], and their transfer to an ambulatory musculoskeletal population undergoing an elective procedure has not been validated.

The practical consequence is a defined research agenda rather than a clinical recommendation. Establishing musculoskeletal-specific reference distributions, in cohorts large enough to support stratification by age and sex, is the first requirement; demonstrating that a pre-procedural index predicts an orthobiologic-specific outcome, rather than a systemic inflammatory marker, is the second. Until both are met, HRV should be treated as a research variable and as a candidate stratification covariate to be tested prospectively, not as a decision rule for selecting or deferring treatment. Framing it otherwise would attribute to the measure a discriminative performance that no published study in this clinical context has yet estimated.

### 6.5. Implications for Trial Design

The framework advanced here is falsifiable, and the design features required to test it are specifiable in advance. A trial capable of doing so would prospectively stratify candidates for orthobiologic injection by pre-procedural vagally mediated HRV and by baseline systemic inflammatory markers, with the stratification variable and its cut-points fixed in the protocol rather than derived from the observed data. A neuromodulatory arm is the natural comparator, and taVNS is the most plausible modality, given a randomized controlled trial reporting pain modulation in knee OA [61], preclinical evidence in post-traumatic OA [120], and its position within the neuromodulation strategies proposed as potentially disease-modifying in OA [106]. The comparator should be a credible sham rather than no intervention, since expectancy effects on pain outcomes are substantial in this population, and blinding success should be measured rather than assumed. Assignment concealment, pre-registration, and a pre-specified primary endpoint are not optional refinements here, because the framework predicts a modest conditioning effect that is precisely the size most vulnerable to bias.

The choice of endpoints determines whether such a trial tests the mechanism or only its clinical shadow. Patient-reported pain and function scores remain necessary as the outcomes that matter to patients, but on their own they cannot distinguish a genuine change in the tissue microenvironment from an analgesic effect of vagal stimulation acting through central pain pathways, which is the interpretation the existing taVNS pain data [61] most directly support. Discriminating between these possibilities requires mechanistic readouts from the joint itself, specifically synovial macrophage M1 to M2 ratios and MMP concentrations in aspirate, together with serial HRV to confirm that the intervention moved the autonomic variable it was intended to move. Including a target-engagement measure is what separates a test of this framework from a conventional efficacy trial, since a negative clinical result with unchanged synovial phenotype and unchanged HRV would indicate failed engagement rather than a false hypothesis. Sample size should be powered on the mechanistic endpoint where feasible, and the analysis should pre-specify how a discordance between molecular and symptomatic outcomes will be interpreted.

Two design decisions deserve explicit attention because they encode the temporal logic set out in Section 5. The timing of conditioning relative to injection is itself a hypothesis and not a nuisance parameter, so a factorial or dose-finding component that varies the pre-procedural conditioning window would yield more information than a single arbitrary interval. Follow-up should extend beyond the interval over which orthobiologic pain relief is typically reported, since the framework predicts an effect on tissue trajectory rather than on early symptom relief, and a short horizon would be structurally incapable of detecting the outcome of interest. A trial constructed along these lines would resolve three separable questions: whether HRV functions as a valid stratification variable in regenerative practice, whether taVNS measurably alters the synovial immunological context, and whether that alteration translates into orthobiologic outcomes. The molecular architecture supporting these predictions is reasonably well characterized, whereas the clinical evidence base remains to be constructed.

### 6.6. A Synthetic Framework and Its Testable Predictions

Taken together, the literature reviewed here supports a synthetic framework in which vagal tone acts as an upstream regulator of the musculoskeletal regenerative microenvironment through activation of the cholinergic anti-inflammatory pathway. Within this framework, α7nAChR engagement shifts macrophage functional states toward pro-resolution (M2-like) programs, restrains NF-κB and NLRP3-dependent transcription of MMPs, and lowers osteoblast RANKL production, a change predicted but not yet shown to move the RANKL/OPG ratio away from osteoclastogenesis, thereby supporting mesenchymal stromal cell engraftment and differentiation and establishing a biologically permissive niche in which orthobiologic interventions such as PRP, BMAc, and MSC-based therapies may act more effectively. Framed in this way, vagal modulation, whether assessed through HRV-based stratification or actively engaged through bioelectronic or pharmacological means, may represent an underrecognized co-therapeutic target in chronic musculoskeletal disease. The framework is offered as a synthesis of convergent evidence rather than as a validated clinical model, and its principal value lies in the specificity of the predictions it generates.

This synthesis is, in principle, testable, in that it predicts that (i) pre-procedural vagal tone, indexed by HRV, will correlate with orthobiologic treatment response, and (ii) experimentally augmenting vagal activity around the time of intervention, through tVNS, iVNS, or pharmacological α7nAChR agonism, will improve regenerative outcomes relative to standard care. Both predictions are falsifiable with existing instrumentation and do not require novel molecular entities. They are precisely the predictions that the prospective trial designs outlined in Section 6.5 are positioned to evaluate. A negative result for either prediction would meaningfully constrain the framework, which is a property that distinguishes it from broader assertions about neuroimmune influence on tissue repair.

## 7. Conclusions

The evidence reviewed here supports a coherent and clinically relevant proposition: vagal tone is positioned by the convergent mechanistic evidence as a plausible regulator, rather than a passive correlate, of the biological terrain within which musculoskeletal repair proceeds or fails; whether it is a determinant of that terrain in patients is the question this framework is intended to make testable. Through the cholinergic anti-inflammatory pathway and the convergent actions of α7nAChR signaling on macrophages, chondrocytes, osteoblasts, synoviocytes, and mesenchymal stromal cells, the nervous system exerts regulatory influence over the principal cellular and molecular axes that govern regenerative outcomes, namely macrophage polarization, NF-κB and NLRP3-driven transcription, MMP-mediated extracellular matrix turnover, RANKL-dependent bone remodeling, MSC viability, and the resolution of chronic synovial inflammation. The molecular specificity of these convergences is what distinguishes this literature from more general accounts of neuroimmune regulation. It is also what makes the framework amenable to direct experimental interrogation, since each node named above corresponds to an established, measurable readout in synovial tissue or fluid.

This framework reinterprets a longstanding clinical observation, namely the unpredictable inter-patient variability in orthobiologic outcomes, as a measurable biological problem. Pre-procedural vagal tone, indexed through heart rate variability, and the inflammatory setpoint of the synovial microenvironment emerge as candidate stratification variables that are quantifiable and potentially modifiable rather than confounders to be managed statistically. The implication for clinical practice is concrete but conditional: neuromodulatory strategies, and transcutaneous auricular vagus nerve stimulation in particular, constitute mechanistically grounded co-therapeutic candidates for patients undergoing PRP, bone marrow aspirate concentrate, or MSC-based interventions, pending prospective evaluation. Their adoption outside of a research setting is not supported by the evidence reviewed here.

Translating this framework into evidence requires prospective trials with HRV-based patient stratification, neuromodulatory arms, and outcome measures that extend beyond symptom scores to include synovial immunophenotyping and longitudinal autonomic monitoring. Molecular endpoints deserve particular emphasis, since the framework makes specific predictions about NF-κB and NLRP3 activity, MMP levels, and the RANKL/OPG ratio that are measurable in synovial fluid and tissue. Such endpoints would allow the mechanism to be tested directly rather than inferred from symptom trajectories. The molecular architecture is reasonably well established; the clinical validation remains to be constructed.

Ultimately, the vagus nerve represents a physiological interface between the central nervous system and the regenerative biology of joints and soft tissues that regenerative medicine has not yet systematically engaged. Engaging it may refine existing orthobiologic protocols, and it may also help address a central unresolved problem in the field, namely why the same biological product, delivered with equivalent technique, produces markedly different outcomes in biologically distinct patients. Whether cholinergic tone accounts for a substantial share of that variance is unknown, and the question is answerable with instrumentation and endpoints that are already available. Establishing the answer, in either direction, would advance the field more than the continued refinement of product composition alone.

## Figures and Tables

**Figure 2:**
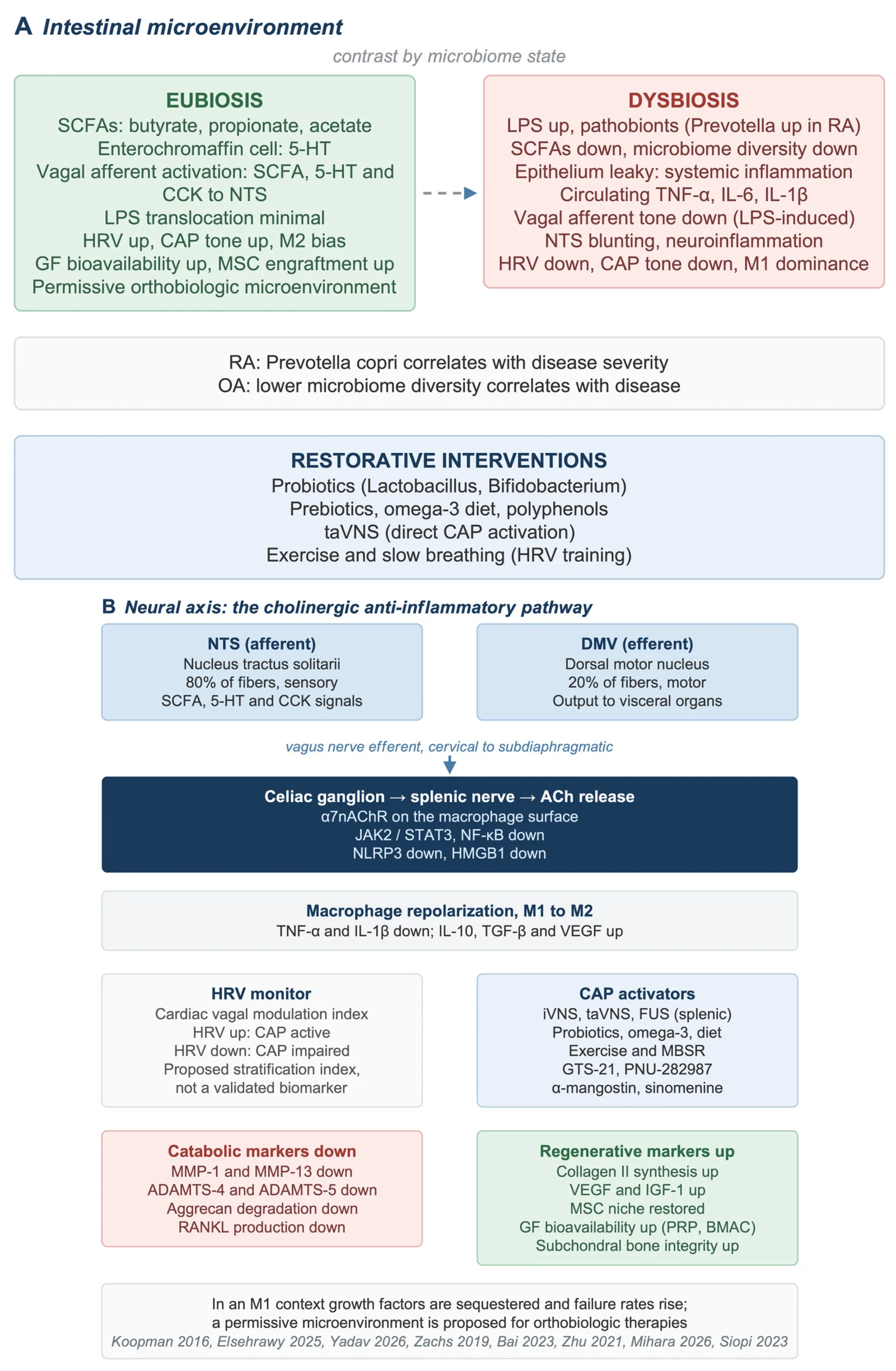

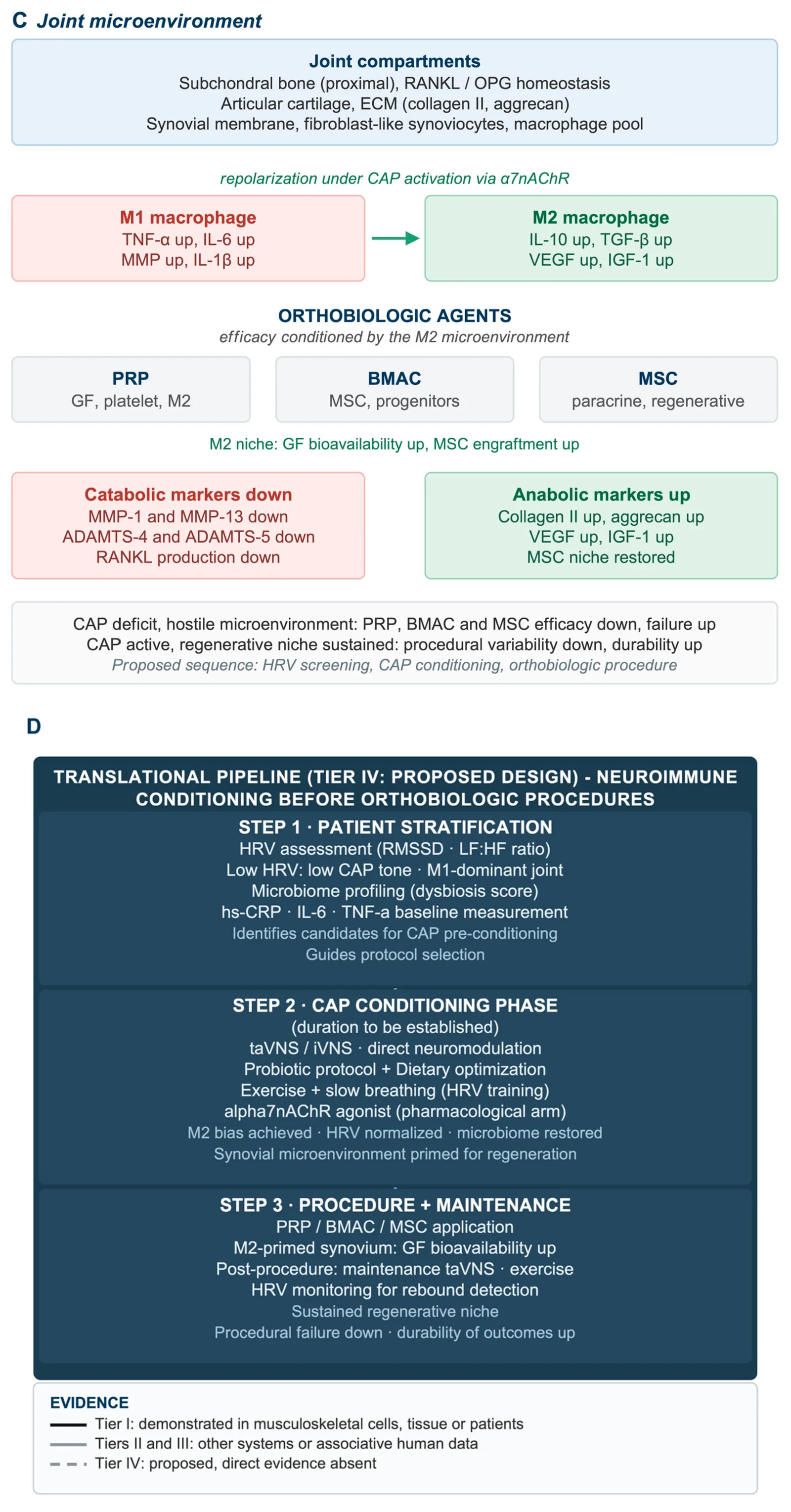
The gut, vagus and joint axis as a systemic modulator of the musculoskeletal microenvironment, presented in four lettered panels across the following pages. (**A**) Intestinal microenvironment. A eubiosis compartment (intact barrier, short-chain fatty acid production, higher HRV and CAP tone with M2 bias) is contrasted against a dysbiosis compartment (interrupted tight junctions, increased lipopolysaccharide and pathobiont load, circulating TNF-alpha, IL-6 and IL-1beta, lower HRV and CAP tone with M1 dominance), closing with a strip of restorative interventions. The enteroendocrine signals and named taxa orient the reader to the wider literature and are not developed here. (**B**) Neural axis. The afferent and efferent vagal limbs are shown from the brainstem nuclei to the coeliac ganglion and the splenic nerve, with acetylcholine release acting on the α7nAChR of macrophages and the downstream suppression of NF-κB and NLRP3. The cardiac vagal index panel is a proposed pre-assessment measure and is not a validated biomarker; see Section 6.4. Connector styles follow the evidence key of panel (**D**). (**C**) Joint microenvironment. The joint compartments are listed in the opening box, from subchondral bone to the synovial membrane, and the panel then contrasts the M1 and M2 macrophage pools and places platelet-rich plasma (PRP), bone marrow aspirate concentrate (BMAC) and mesenchymal stromal cell preparations in a niche whose efficacy is depicted as conditioned by macrophage phenotype. The two closing strips state the opposing predictions of the framework, namely reduced orthobiologic efficacy under cholinergic deficit and a sustained regenerative niche under active cholinergic tone; both are predictions of the hypothesis and not demonstrated outcomes. (**D**) Proposed translational pipeline for neuroimmune conditioning before orthobiologic procedures, in three steps: stratification by HRV, microbiome profiling and baseline inflammatory markers; a pre-procedural CAP conditioning phase, labelled in the artwork as of a duration to be established; and the procedure with maintenance monitoring. No clinical data support any particular duration for this phase, and no numerical interval is printed in the artwork. The entire panel is Tier IV of Table 1 and is drawn accordingly, as the evidence key at the foot records. This pipeline is a testable design and not validated clinical practice. The RMSSD and LF:HF indices orient the reader to the wider literature and are not developed in this review. The figures were conceptualized by the authors based exclusively on the cited primary literature; the tool used in their preparation is declared in the Acknowledgments [15,23,27,34,36,61,62,120].

**Table 1:** Classification of the principal claims of this review by evidence tier. The claim, the tier and the basis for that tier are given in separate columns, so that the grading of each claim and the qualification attached to it are read independently. Two claims are recorded as not established rather than assigned a tier, because the supporting data do not exist. The central proposition of this review, that cholinergic tone conditions the microenvironment in which orthobiologics act, is Tier IV, and no argument developed in the text upgrades it.

Claim	Evidence Tier	Basis and Qualification
α7nAChR expression and function in chondrocytes, osteoblasts, synoviocytes, and macrophages	Tier I	Demonstrated in musculoskeletal cells, tissue, or patients
α7nAChR activation suppresses NF-κB and NLRP3 signaling in joint-resident cells	Tier I	Demonstrated in musculoskeletal cells, tissue, or patients
α7nAChR activation reduces MMP-1, MMP-3, and MMP-13 expression in chondrocytes	Tier I	Demonstrated in musculoskeletal cells, tissue, or patients
Reduced RANKL production by osteoblasts after α7nAChR activation	Tier I	Demonstrated in musculoskeletal cells, tissue, or patients
Implantable vagus nerve stimulation reduces disease activity and TNF-α in rheumatoid arthritis	Tier I	Demonstrated in musculoskeletal cells, tissue, or patients
Vagotomy accelerates osteoarthritis progression in rodent models	Tier I	Demonstrated in musculoskeletal cells, tissue, or patients
MSC cholinergic receptor repertoire and modulation of migration and paracrine output	Tier I	Demonstrated in musculoskeletal cells, tissue, or patients
MSC anti-inflammatory effect requires an intact vagal arc	Tier II	Demonstrated in ARDS and sepsis models, not in joint tissue
Cholinergic priming of platelet α-granule release	Tier II	Demonstrated outside orthobiologic preparations
Vagal dependence of gut-derived inflammatory signaling	Tier II	Demonstrated in other systems; target independently shown in musculoskeletal tissue
Low heart rate variability associates with elevated CRP and IL-6	Tier III	Associative human data only
Effect of α7nAChR activation on OPG and on the RANKL/OPG ratio under vagal stimulation	Not established	Data do not exist; no tier assigned
HRV as an index of α7nAChR-dependent inflammatory reflex activity in the joint	Not established	Data do not exist; no tier assigned
Vagal conditioning improves PRP, BMAc, or MSC outcomes	Tier IV	Proposed; no prospective trial has tested it
Duration of a pre-procedural conditioning phase	Tier IV	Proposed design parameter; no clinical data support any duration
HRV-based patient stratification before orthobiologic delivery	Tier IV	Proposed experimental design, not a clinical pathway

**Table 2:** Comparative matrix of interventions that engage the cholinergic anti-inflammatory pathway (CAP) in musculoskeletal disease. Nine interventions are listed with modality, primary mechanism, downstream effects and clinical application. This content was presented as a graphical matrix in the submitted version; it is rendered here as a table so that every entry scales with the body text and remains legible at any page size. The evidence tier and the translational limitations of each intervention are given in Table 3, where the tier assigned to each row follows the classification of Table 1 and each entry states what remains untested. Two clarifications are warranted. First, inclusion in this matrix denotes that a modality engages the pathway, not that it is a plausible clinical strategy: the selective agonists PNU-282987 and GTS-21 appear as mechanistic probes, and the reasons why nicotine is not a candidate therapy are given in Section 3.2. Second, rows differing by two or more evidence tiers in Table 3 should not be read as alternatives to one another. All entries were synthesized by the authors exclusively from the cited primary literature.

Intervention and Modality	Primary Mechanism	Downstream Effects	Clinical Application
Implantable VNS (iVNS). Bioelectronic; Cervical surgical implant	Cervical vagal efferent activation; to celiac ganglion to splenic arc; to α7nAChR on splenic macrophages; JAK2/STAT3 increased; NF-κB decreased	TNF-α decreased; IL-1β decreased; DAS28 decreased; Macrophage M2 polarization increased; Synovial inflammation decreased; Platelet α-granule release primed	Rheumatoid arthritis; Drug-resistant epilepsy; Treatment-resistant depression
Transcutaneous auricular VNS (taVNS). Auricular branch stimulation	Auricular branch to NTS/DMV; to efferent CAP activation; to peripheral macrophage M2; α7nAChR; NF-κB decreased; NLRP3 decreased	IL-6 decreased; CRP decreased; pain (VAS) decreased; WOMAC increased (knee OA RCT); Cartilage/bone destruction decreased (CIA); Post-traumatic OA pain decreased	Knee OA (human RCT); Post-traumatic OA; RA (animal models); Pre-procedural CAP priming
Focused ultrasound, splenic stimulation (FUS). Bioelectronic, non-invasive	Non-invasive splenic arc activation; via focused ultrasound; to α7nAChR on macrophages; No surgical implant required	TNF-α decreased; CIA disease score decreased; ≈pharmacological CAP activation; Splenic immune modulation increased	Inflammatory arthritis (preclinical); Non-invasive iVNS alternative; Future: OA, RA clinical trials
GTS-21 (α7nAChR partial agonist). Synthetic; pharmacological	Selective α7nAChR partial agonist; to JAK2/STAT3 to NF-κB decreased; Peripheral monocyte trafficking decreased; Synovial infiltration inhibited	Synovial monocyte infiltration decreased; CIA disease severity decreased; M1 cytokine profile decreased; Macrophage M2 skewing increased	RA (preclinical); Pre-procedural immunomodulation; (proposed)
PNU-282987 (selective α7nAChR agonist). Synthetic; pharmacological	Full α7nAChR agonist to NLRP3 decreased; NF-κB decreased; autophagy increased; MMP-1, MMP-13 decreased in chondrocytes; M2 polarization increased (CD206, IL-10)	OA cartilage degeneration decreased; Chondroprotection (histological); Autophagy-mediated protection increased; M2 increased; ECM degradation decreased	OA (preclinical); Chondroprotection research; M2 polarization mechanistic tool
Phytocompounds (α-mangostin, notopterol, sinomenine). Natural CAP agonists	CAP-SIRT1 pathway (α-mangostin); α7nAChR metabolic reprog. (notopterol); α7nAChR increased + NF-κB decreased in FLS; (sinomenine)	CIA/adjuvant arthritis decreased; Synovitis decreased; M1 markers decreased; FLS invasive capacity decreased; ECM degradation decreased	RA (preclinical); Natural CAP activator candidates; FLS-targeted therapy
Dietary interventions (omega-3, polyphenols). Anti-inflammatory dietary patterns	Microbiota modulation to vagal; afferent increased to HRV increased to CAP increased; SCFA-enteroendocrine signaling; Gut–joint axis modulation	CRP decreased; IL-6 decreased (observational); HRV improvement (prospective); LPS translocation decreased; Inflammaging prevention	OA, RA: systemic support; Gut–joint axis modulation; Inflammaging prevention
Probiotic supplementation. Microbiota-directed; Systemic; Gut–vagal axis modulation	Microbiota restoration to tight; junction increased to LPS decreased to vagal; afferent recovery to CAP increased	Systemic inflammation decreased; Gut permeability decreased; Vagal afferent activity increased (animal)	Dysbiosis-associated MSK; RA, OA gut-joint modulation; CAP tone restoration
Exercise, slow breathing, MBSR. Lifestyle; HRV-mediated CAP conditioning	HRV increased to sustained CAP tone increased; to M2 macrophage maintenance; Vagal efferent conditioning; Autonomic rebalancing	IL-6 decreased; TNF-α decreased; pain decreased; (OA/RA RCTs); HRV improvement (prospective); Inflammatory rebound prevention	OA, RA: co-therapeutic; Pre-procedural CAP conditioning; Post-procedural maintenance

**Abbreviations:** CAP, cholinergic anti-inflammatory pathway; CIA, collagen-induced arthritis; DMV, dorsal motor nucleus; ECM, extracellular matrix; FLS, fibroblast-like synoviocytes; GTS-21, 3-(2,4-dimethoxybenzylidene)-anabaseine; HRV, heart rate variability; iVNS, implantable VNS; MBSR, mindfulness-based stress reduction; MMP, matrix metalloproteinase; MSC, mesenchymal stromal cell; NF-κB, nuclear factor-kappa B; NLRP3, NOD-like receptor protein 3; OA, osteoarthritis; RA, rheumatoid arthritis; SIRT1, sirtuin-1; taVNS, transcutaneous auricular VNS; α7nAChR, alpha7 nicotinic acetylcholine receptor.

**Table 3:** Evidence tier, evidence base and outstanding limitations of each intervention listed in Table 2. The tier assigned to each row follows the four-level classification of Table 1. Rows differing by two or more evidence tiers should not be read as alternatives to one another.

Intervention	Evidence Tier	Evidence Base	Key Limitations and Evidence Gaps
Implantable VNS (iVNS)	Tier I	Human open-label trial [62]; (RA, n = 17)	Surgical implantation required; High cost; limited scalability; No OA-specific or orthobiologic trial; Stimulation parameters not standardized
Transcutaneous auricular VNS (taVNS)	Tier I	Human RCT [61]; (knee OA, randomized); animal model [120]	Parameter standardization lacking; Optimal protocol not defined; No orthobiologic protocol integration; Long-term follow-up data limited
Focused ultrasound, splenic stimulation (FUS)	Tier II	Animal model [36]; (CIA murine model)	No human MSK data; Targeting precision unclear; Translation pathway not defined; Device standardization pending
GTS-21 (α7nAChR partial agonist)	Tier II	Animal model [34]; (CIA murine)	Receptor desensitization risk; Blood–brain barrier penetration; No human MSK trial; Dosimetry for joint context undefined
PNU-282987 (selective α7nAChR agonist)	Tier I	Animal + in vitro [23,28]; (OA; THP-1 macrophages)	Context-dep. TFEB pro-inflam. effect; Pharmacokinetics undefined; No human data; Dosimetry critical for M2 directionality
Phytocompounds (α-mangostin, notopterol, sinomenine)	Tier II	Animal model [31,33,49]; (CIA / adjuvant arthritis)	Bioavailability uncharacterized; No human RCT in MSK; Receptor specificity varies by compound; Pharmacodynamics need profiling
Dietary interventions (omega-3, polyphenols)	Tier III	Indirect/observational; Human observational studies; Mechanistic: animal models; No MSK-specific RCT	CAP attribution mechanistically indirect; No MSK-specific intervention RCT; Dose–response uncharacterized; Multiple confounders in observational data
Probiotic supplementation	Tier II	Animal model [15]; (gut–vagus–brain axis); Limited human mechanistic data	Strain specificity critical; Dose–response undefined; MSK-specific outcomes not measured; CAP-mediated mechanism not isolated
Exercise, slow breathing and mindfulness-based stress reduction	Tier III	Human prospective; OA/RA exercise RCTs; HRV-inflammation meta-analysis [91]	CAP attribution indirect; Dose–response for CAP unknown; No orthobiologic-coupled trial; Adherence and access barriers

Abbreviations are as defined in Table 2 and in the abbreviations list.

## Data Availability

No new data were created or analyzed in this study. All data discussed are available in the cited published sources.

## Abbreviations

The following abbreviations are used in this manuscript:

ACh	Acetylcholine
ADAMTS	A disintegrin and metalloproteinase with thrombospondin motifs
BMAc	Bone marrow aspirate concentrate
CAP	Cholinergic anti-inflammatory pathway
ChAT	Choline acetyltransferase
CIA	Collagen-induced arthritis
CRP	C-reactive protein
ECM	Extracellular matrix
FLS	Fibroblast-like synoviocytes
HMGB1	High-mobility group box protein 1
HRV	Heart rate variability
IGF-1	Insulin-like growth factor 1
IL	Interleukin
iVNS	Implantable vagus nerve stimulation
LPS	Lipopolysaccharide
MMP	Matrix metalloproteinase
MSC	Mesenchymal stromal cell
NF-κB	Nuclear factor kappa B
NLRP3	NOD-like receptor protein 3 (inflammasome)
OA	Osteoarthritis
OPG	Osteoprotegerin
PDGF	Platelet-derived growth factor
PGE2	Prostaglandin E2
PRP	Platelet-rich plasma
RA	Rheumatoid arthritis
RANKL	Receptor activator of NF-κB ligand
taVNS	Transcutaneous auricular vagus nerve stimulation
TGF-β	Transforming growth factor beta
TNF-α	Tumor necrosis factor alpha
tVNS	Transcutaneous vagus nerve stimulation
VAChT	Vesicular acetylcholine transporter
VEGF	Vascular endothelial growth factor
VNS	Vagus nerve stimulation
α7nAChR	Alpha7 nicotinic acetylcholine receptor
